# Rapid morphological change in multiple cichlid ecotypes following the damming of a major clearwater river in Brazil

**DOI:** 10.1111/eva.13080

**Published:** 2020-09-23

**Authors:** Michelle C. Gilbert, Alberto Akama, Cristina Cox Fernandes, R. Craig Albertson

**Affiliations:** ^1^ Organismic and Evolutionary Biology Graduate Program University of Massachusetts Amherst MA USA; ^2^ Museu Paraense Emílio Goeldi Belém PA Brazil; ^3^ Instituto Nacional de Pesquisas da Amazônia Manaus Brazil; ^4^ Biology Department Morrill Science Center University of Massachusetts Amherst MA USA

**Keywords:** adaptation, anthropogenic change, contemporary evolution, geometric morphometrics, phenotypic plasticity

## Abstract

While anthropogenic disturbances can have damaging effects on biodiversity, they also offer an opportunity to understand how species adapt to new environments and may even provide insights into the earliest stages of evolutionary diversification. With these topics in mind, we explored the morphological changes that have occurred across several cichlid species following the damming of the Tocantins River, Brazil. The Tocantins was once a large (2,450 km), contiguous river system; however, upon closure of the Tucuruí Hydroelectric Dam in 1984, a large (~2,850 km^2^), permanent reservoir was established. We used geometric morphometrics to evaluate changes in native cichlids, comparing historical museum specimens collected from the Tocantins to contemporary specimens collected from the Tucuruí reservoir. Six species across five genera were included to represent distinct ecomorphs, from large piscivores to relatively small opportunistic omnivores. Notably, statistically significant changes in shape and morphological disparity were observed in all species. Moreover, the documented changes tended to be associated with functionally relevant aspects of anatomy, including head, fin, and body shape. Our data offer insights into the ways cichlids have responded, morphologically, to a novel lake environment and provide a robust foundation for exploring the mechanisms through which these changes have occurred.

## 1 INTRODUCTION

Anthropogenic alterations to an ecosystem can provide opportunities for studying how populations respond to rapid ecological change. Over the past 80 years, there has been mounting evidence that modifications to waterways can have immediate, and lasting, ecological consequences that may result in varying degrees of habitat destruction and fragmentation (Allan & Flecker, 1993; Geladi et al., 2019; Helfman, 2007; Lau, Lauer, & Weinman, 2006; Trautman, 1939). Dam and reservoir construction, in particular, can have long reaching ecological effects on fish communities (Agostinho, Pelicice, & Gomes, 2008), including shifts in local fish assemblages (Platania, 1991), impeding the movement of migrating species (Dugan et al., 2010; Liermann, Nilsson, Robertson, & Ng, 2012) and disrupting the reproductive success of species that depend on the flow of water for egg dispersal (Alò & Turner, 2005; Platania & Altenbach, 1998). Further, there is significant concern that such drastic changes increase the risk of extirpation or extinction of local populations, due to reduced genetic diversity (Alò & Turner, 2005; Higgins & Lynch, 2001; Nei, Maruyama, & Chakraborty, 1975).

With the human population continuing to increase, so too is the demand for energy. To meet these needs, there is an increasing reliance on exploiting lotic freshwater systems, with over 3,700 dams either currently under construction or planned, many of which are in developing countries (Winemiller et al., 2016; Zarfl, Lumsdon, Berlekamp, Tydecks, & Tockner, 2014). The Tocantins River is one such system that is increasingly being utilized to meet the energy demands of a growing population. Historically characterized by fast flowing rapids, sand and rock covered waterbeds, and rich seasonal floodplains (de Mérona, 1987), the Tocantins is one of the largest clearwater rivers in South America. With a total length of roughly 2,450 km, the Tocantins River passes through four Brazilian states and serves as the major drainage for both the Tocantins and Araguaia watersheds. Over the past 30 years, the Tocantins River has seen increased anthropogenic activity, including deforestation, agriculture, and the construction of numerous dams. The Tucuruí Hydroelectric Dam is the largest and oldest of these, and its construction has resulted in the establishment of a large (2,850 km^2^) permanent reservoir, stretching nearly 70 km in length and 40 km wide (ELETROBRÁS/DNAEE, 1997).

Since the completion and subsequent closure of the Tucuruí Hydroelectric Dam in 1984, the Tocantins River has experienced drastic hydrological, ecological, and geomorphological changes (Fearnside, 2001). For instance, the once historic rapids and streams that characterized the system have disappeared from the surrounding area, which in turn has affected the abundance and variety of food sources available to native fishes (Araújo‐Lima, Agostinho, & Fabré, 1995). Alterations such as these may create opportunities for fishes that are inclined to be trophic generalists (Angermeier, 1995; Wilson et al., 2008), while greatly impairing trophic specialists that rely on specific habitats for various life stages, behaviors, and feeding ecologies. Changes in the local fish assemblages within the reservoir have already been observed, including a reported 20% reduction in species diversity since 1984 (Santos, Jégu, & de Mérona, 1984; Santos, de Mérona, Juras, & Jégu, 2004).

Habitat destruction and fragmentation are a significant threat to biodiversity (Vitousek, Mooney, Lubchenco & Melillo, 1997), and such rapid ecological alterations require populations to adapt or face local extinction. Indeed, habitat fragmentation can spur the contemporary evolution of populations and has even been shown to result in increased speciation and rapid genetic divergence in natural systems (Dias, Cornu, Oberdorff, Lasso, & Tedesco, 2013). Generally speaking, fishes are well known to be capable of quickly responding to rapid ecological shifts (Collyer, Hall, Smith, & Hoagstrom, 2015; Hulsey, Hendrickson, & García de León, 2005; Rüber & Adams, 2001), including anthropogenic change (Candolin, 2009; Franssen, 2011; Franssen, Harris, Clark, Schaefer, & Stewart, 2013). However, there remains a pressing need to evaluate native fish species that have been subjected to such rapid changes to assess if, how, and why populations respond to sudden human‐induced change.

Cichlids (Cichliformes:Cichlidae) are a highly diverse and well‐studied family of fishes that have repeatedly undergone extensive adaptive radiations (Arbour & López‐Fernández, 2013; López‐Fernández, Honeycutt, & Winemiller, 2005), and are well known to adapt quickly to ecological change, especially those that influence the foraging environment (Muschick, Barluenga, Salzburger, & Meyer, 2011; Wimberger, 1991, 1992). Native cichlid populations in and around the Tocantins River and Tucuruí reservoir exhibit a wide range of life‐history strategies, breeding, and food preferences. Such diversity allows for the exploration and assessment of several foraging ecomorphs, from large, piscivorous predators to small mud sifters. This system, and the alterations it has experienced, provides a unique opportunity to examine how large‐scale alterations to an aquatic system can spur incidents of rapid adaptation in complex fish communities.

In this study, we compared the morphology of six cichlid species collected from the Tucuruí reservoir in 2018 (postdam) to that of cichlids collected at the same locality prior to or just after the closure of the Tucuruí Hydroelectric Dam in 1984 (predam). Our primary aim is to acquire insights into if, and how, the cichlid community has adapted, in terms of shape, during the past 34–48 years to this novel environment. While traditional morphometrics can provide a direct and intuitive link to specific, functionally relevant characters, species rarely change in just one trait, and adaptation often involves subtle, coordinated shifts across a suite of characters (Adams, Rohlf, & Slice, 2004; Rohlf & Marcus, 1993). By focusing on geometry, geometric morphometric complements and extends traditional linear measures to provide a more holistic understanding of variation in organismal shape (Zelditch, Swiderski, Sheets, & Fink, 2012). Further, by using species that represent different ecotypes we will be able to assess the extent to which feeding behavior and ecology can predict the magnitude or pattern of anatomical change. Our hope is that this study will provide insights as to how species respond to rapid environmental change, especially anthropogenic change, and how it may affect the Amazon.

Our overarching hypothesis is that the formation of the Tucuruí reservoir has induced shifts in habitat and foraging behavior and concomitant changes in the anatomy of resident cichlid populations as they adapt to novel environmental conditions. This study represents a first step toward assessing this hypothesis. Given the varied ecologies, foraging behaviors, and life histories of species included in our analysis, a number of more specific predictions arise from this study. *Cichla* are large piscivorous fishes, capable of easily navigating a lotic system. Proposed similarities between *C. kelberi* and *C. pinima* included prognathous lower jaws and maxillae that extended below the middle of the orbit. We expected contemporary specimens of *Cichla* to converge on a phenotype that includes deeper heads and bodies to reflect the change to a large, deep lentic system (Collin & Fumagalli, 2011; Gaston & Lauer, 2015). Our analyses also included four smaller species: two foraging generalists (*G. neambi* and *S. jurupari* **)** and two specialists (*C. spectabilis* and *H. efasciatus*). Broadly speaking, habitat degradation and global change are drivers of the observed replacement of specialists by generalists (Clavel, Julliard, & Devictor, 2011). In principle, generalists should be more resilient than specialists to ecological changes that influence resource availability (Vázquez & Simberloff, 2002), a scenario that has been widely observed in numerous taxa, including fishes (Angermeier, 1995; Wilson et al., 2008). Thus, we predicted that *G. neambi* and/or *S. jurupari* would exhibit changes in shape and potentially morphological disparity as a result of the introduction of new niche space from flooded riparian and altered hydrology. *C. spectabilis* are ambush predators that consume a variety of prey items including arthropods and smaller fishes. *H. efasciatus* also consume a variety of food items but are inclined toward being a frugivore. Given the relatively narrow feeding repertoires of these two species, we expected to observe less conspicuous changes in their shapes.

## 2 MATERIALS AND METHODS

### 2.1 Study site

**FIGURE 1:**
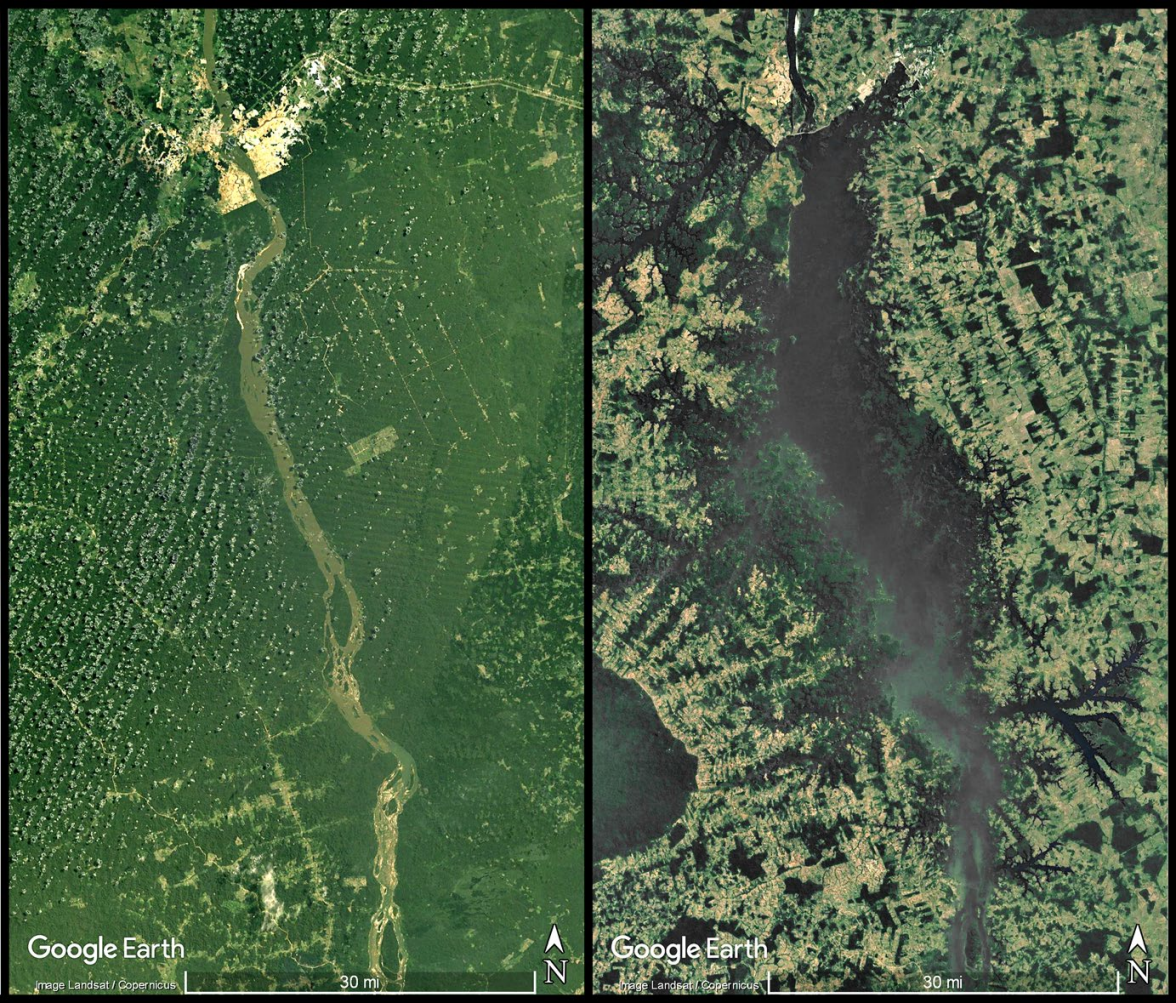
Google Earth imagery (Landsat/Copernicus) of Tucuruí Hydroelectric dam vicinity, before (left; December 1984) and after (right; December 2016) dam construction. Scale bar represents ~48.3 km (30 miles)

The Tocantins River presents several dams along its course, such as the Serra da Mesa and Lageado, but the Tucuruí is the largest with the most substantial impact (Akama, 2017). The Tucuruí reservoir (Figure 1; Appendix S1) covers more than 2.875 km^2^, 400 km upstream from the river mouth (de Mérona, Mendes Dos Santos, & Goncçalves de Almeida, 2001; Santos et al., 2004). Its shape is dendritic with a maximum depth of 75 m close to the dam and a mean depth of 17 m, with some shallow areas that can become exposed during the dry season (dos Santos et al., 2004). Fish inventories were conducted prior to closure in 1980–1982, specimens from which are housed in the *Instituto Nacional de Pesquisas da Amazônia* (INPA) fish collection. Before this, during the 1970s, expeditions in Pará state (Expedição Permanente na Amazônia, “Permanent Expedition to Amazon”) also collected in pristine areas of the Tocantins River, with specimens housed at the *Museu de Zoologia da Universidade de São Paulo* (MZUSP). Many of these specimens were used in this study.

### 2.2 Fish collections

To investigate whether native cichlid populations have experienced a rapid change in trophic and body shape morphology, we compared museum specimens (Table S1) collected in the region ≤1984 to fishes collected from the Tucuruí reservoir in 2018. However, *Cichla pinima* samples were from 1987, collected shortly after the dam was closed, and just as the reservoir appears to have reached its current size (Figure S1). Issues for comparing fixed museum specimens with fresh specimens were considered. However, such comparisons are not uncommon in evolutionary studies and while fixation can influence overall specimen size, the effects on shape have been shown to be minimal (Gaston & Lauer, 2015; Larochelle, Pickens, Burns, & Sidlauskas, 2016). Further, the aspects of shape we assessed tended to be associated with specific bony elements (e.g., jaw length, orbit size, anal fin insertion) that would presumably be less prone to the effects of fixation. Contemporary specimens were obtained fresh from the Association of Fisherman in Tucuruí and directly from fishermen at the margin of Tucuruí reservoir, March 2018, and imaged in the field, apart from *Geophagus neambi* which were collected in January of 2018. Specimens were obtained from multiple fisherman and, therefore, came from multiple collection events around the reservoir. Since nearly all contemporary specimens were collected from local vendors, viscera (including gonads) were often not present, making it impossible to include sex as a trait in our study. These specimens were accessioned in the fish collection at Museu Paraense Emílio Goeldi (MPEG) in Belém, PA, Brazil. In total, we examined six species across five genera that encompassed a range of trophic ecologies.

### 2.3 Study taxa (Ecology)

*Cichla kelberi* and *C. pinima*. The peacock basses are large, piscivorous fishes that are commercially important and widely distributed throughout the Amazon basin. *Cichla* species have been reported to rapidly colonize, and undergo population expansion, within dammed sections of rivers (Santos, 1995; Santos & Oliveira Jr., 1999). This is likely because they will readily consume a wide variety of fish species (Fugi, Luz‐Agostinho, & Agostinho, 2008; Novaes, Caramaschi, & Winemiller, 2004; Williams, Winemiller, Taphorn, & Balbas, 1998). Further, while cannibalism is a common phenomenon in this genus (e.g., *C. monoculus, C. kelberi*), it has been reported to occur more frequently in systems affected by dam operation (Novaes et al., 2004; Rosemara Fugi et al., 2008).

*Geophagus neambi. G. neambi* is a recently described species (Lucinda, Lucena, & Assis, 2010) from the Tocantins drainage. Across the genus *Geophagus*, a range of reproductive strategies are utilized, including mouthbrooding (e.g., *G. steindachneri*) and substrate spawning (e.g., *G. brasiliensis*), the former which may create functional constraints on how trophic morphology is allowed to change. The diet of certain *Geophagus* species (i.e., *G. brasiliensis*) suggests a generalist/opportunistic diet that includes gastropods, vegetation, and fish scales (Bastos, Condini, Varela, & Garcia, 2011). Previous studies have shown *Geophagus* species to be phenotypically plastic, both in terms of jaw and body shape morphology, when presented with different diets (Wimberger, 1991, 1992). Further, a related species, *G. surinamensis* (possibly misidentified *G. neambi*), was found to have shifted from an ancestrally omnivorous diet to one more characteristic of a detritivore, whereas within the Tucuruí reservoir this species appeared to adopt an unspecialized carnivorous diet (de Mérona et al., 2001).

*Satanoperca jurupari*. Formerly named *Geophagus jurupari*, *S. jurupari* is widely distributed throughout the Amazon Basin and is generally characterized by a more omnivorous diet. In the Lake Tucuruí system, *S. jurupari* was classified as a broad invertivore prior to the closure of the Tucuruí Hydroelectric Dam, but has since been found to consume more of an omnivore diet both within Lake Tucuruí and downstream of the dam (de Mérona et al., 2001). Reproductive behavior includes mouth brooding following ~24 hr of substrate spawning (Reid & Atz, 1958). Further, parents have been observed to actively move nest sites in response to changing water levels for the first 24 hr postspawning (Cichocki, 1976). Much like *Geophagus,* mouth brooding behavior in this genus may create constraints on the functional aspects of trophic morphology and the degree to which it can respond to different feeding regimes.

*Caquetaia spectabilis. Caquetaia* species are carnivorous predators, well known for their extreme jaw protrusion, which is reported to be an adaptation that enhances foraging efficiency on elusive prey (Waltzek & Wainwright, 2003). Altered hydrologic cycles have been reported to influence the activity and feeding behavior of *C. spectabilis*, notably during dry or low periods where individuals seem to become increasingly sedentary and feed on a greater variety of food items (Röpke, Ferreira, & Zuanon, 2014).

*Heros efasciatus. H. efasciatus* is an omnivore, and one of few cichlid species that acts a frugivore, especially during flood periods when it is able to exploit riparian zones and floodplains (Favero, dos Pompeu, & Prado‐Valladares, 2010). *H. efasciatus* spawn during October and December, presumably so that the eggs will hatch during the flood season (Favero et al., 2010). Fecundity of individuals has been reported to be low (Favero et al., 2010), suggesting that populations could be highly susceptible to rapid ecological change.

### 2.4 Data collection

We photographed the left‐lateral surface of museum and field collected specimens using a tripod and digital camera (Olympus E520). A scale bar was included alongside each photograph to later scale landmark data, and dorsal, caudal, anal, and pelvic fins were pinned when they obscured the body profile. A range of sizes were available from museum collections, whereas sizes from freshly collected specimens tended to be larger (Table S1). Since sample sizes from the museum were often limited, all museum specimens in workable condition were included, and size effects were later corrected for mathematically (see below). Here, workable condition is defined as lacking conspicuous bending and severe tissue deformation. Total sample sizes for all species:year groups are given in figure legends.

**FIGURE 2:**
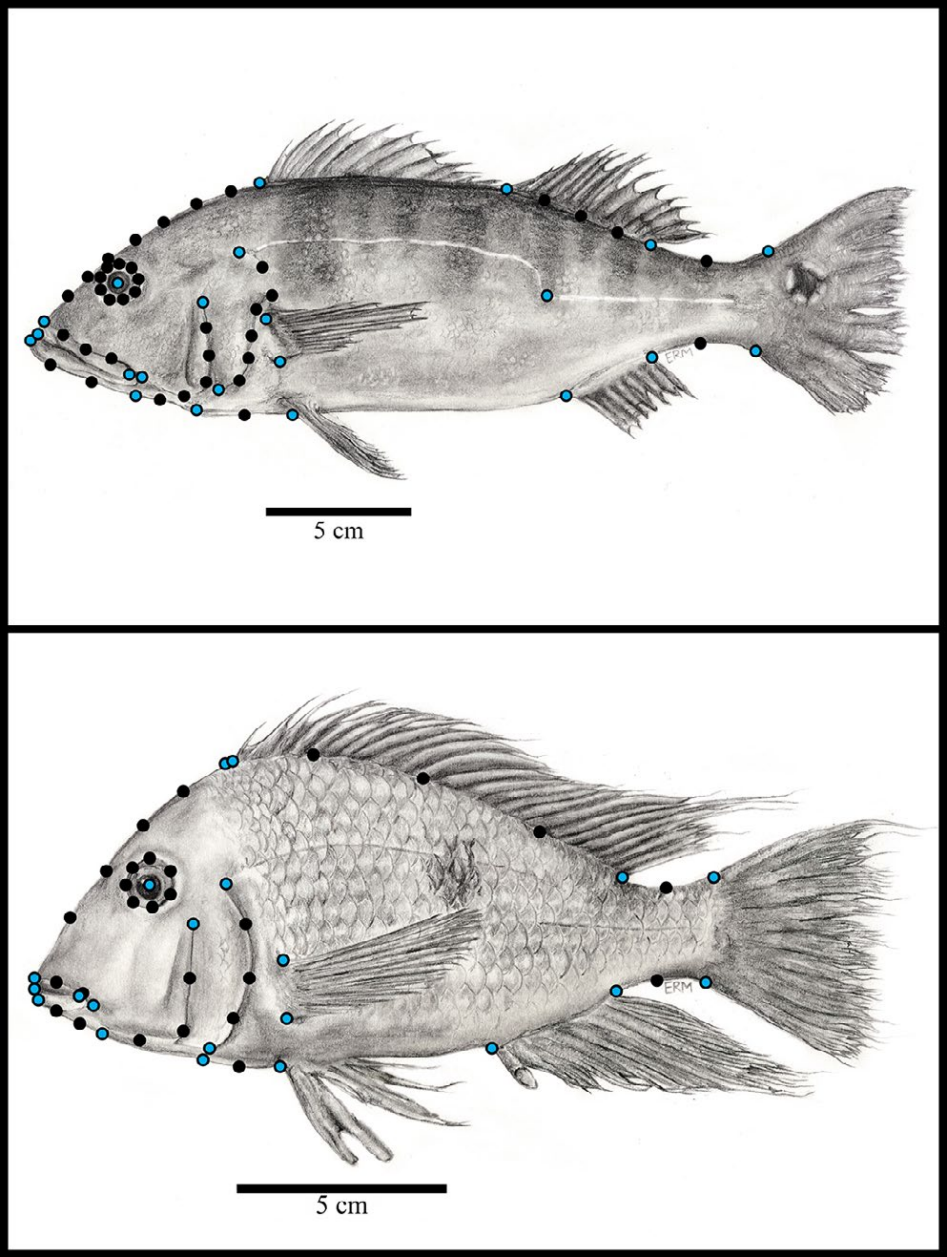
Anatomical landmarks used for geometric morphometric analysis. Large, blue circles represent fixed landmarks, small black circles represent semi/sliding landmarks. *Cichla* (top). A total of 60 landmarks were used, 22 of which were fixed landmarks, including one fixed landmark at the beginning of the second lateral line. Other cichlids (bottom). A total of 48 landmarks were used, 21 of which were fixed landmarks. Fixed landmarks include premaxillary groove, end of nape, insertion of dorsal fin (second dorsal for *Cichla*), end of dorsal fin, upper and lower ends of the caudal peduncle, insertion and end of anal fin, beginning of breast and insertion of pelvic fin, pectoral fin insertion, opercle & preopercle beginning and end, lower jaw insertion and anterior tip, tip and posterior end of premaxilla, posterior end of maxillary groove, center of the eye, and insertion of the lower lateral line (*Cichla* only). Illustrations hand‐drawn by Emma R Masse

Landmark configurations for *Cichla* consisted of 22 “fixed” anatomical landmarks and 38 sliding semilandmarks. For the remaining species, the landmark configurations consisted of 21 “fixed” anatomical landmarks and 27 sliding semilandmarks (Figure 2). We used a greater number of landmarks in *Cichla* because they possess two distinct dorsal fins, as opposed to the more continuous dorsal fins seen in the remaining four genera. They also exhibited interesting patterns of variation in the positioning of the lateral canal along the body that we wished to quantify. While fixed landmarks correspond to homologous anatomical points of interest, semilandmarks allow for more nuanced variation found in complex structures, such as curves, to be “captured” and quantified. Being allowed to slide along curves during a generalized Procrustes analysis (GPA; Rohlf & Slice, 1990), semilandmarks allow for the minimizing of Procrustes distances between landmarks of curves and permit one to quantify the curvature of complex structures (Gunz & Mitteroecker, 2013). Landmarking provided X, Y Cartesian coordinates that we used to generate Procrustes residuals during GPA. GPA works by mathematically removing the effects of size, scale, and orientation to allow for one to determine mean shapes (Goodall, 1991) to generate said Procrustes residuals around the mean that reflect specimen variation. All specimens were digitized using STEREOMORPH (Olsen & Westneat, 2015).

In addition to collecting 2‐D geometric morphometric data, we also collected four linear measures across all specimens, predam and postdam. This was done as a supplement of our geometric morphometric data to assess possible changes in specific traits with explicit ecological relevance. Linear measurements were taken digitally using TPSdig software (v2.2; Rohlf, 2016) and consisted of body depth (indicator of maneuverability and swimming speed), jaw length (relevance to prey capture mechanism and preference), caudal peduncle length (indicator of swimming performance, e.g., cruising or noncruising), and eye diameter (pertinent to prey preference). Figure S2 illustrates how and where each measurement was taken. We calculated slopes from linear measurements within species groups and used ANOVA on least squared means to compare pre/postdam values, allowing us to determine whether trajectories were parallel or divergent. We also corrected for allometric effects via linear regression where linear traits were the dependent variable and standard length was the predictor variable. Residuals were subjected to Welch two‐sample *t* tests to test for pairwise significance (predam versus postdam) for all species, with the exception of *Cichla* which we subjected to a Tukey honest significant difference test in order to make comparisons between the two species and year collected. While the comparison of residual, size‐corrected values assumes that slopes are parallel (McCoy, Bolker, Osenberg, Miner, & Vonesh, 2006), our sampling scheme was not intended to describe and compare allometric trajectories. Specifically, we only sampled a limited range of age/sizes, with an emphasis on older fishes, and therefore, we are careful not to overinterpret these results. Accordingly, we chose to proceed with such comparisons even in the few cases where slopes were statistically divergent, but encourage caution when interpreting these results. In general, we consider the linear measures to be a supplement to the geometric morphometric methods.

### 2.5 Geometric morphometric analyses

GPA‐aligned coordinate data were subjected to various statistical tests to assess differences between ≤ 1984 collections and our 2018 collection. Comparisons of shape were performed within cichlid genera. We chose this taxonomic level, as cichlid genera are generally defined by ecomorphological differences. *Cichla* was the only genus containing two species, and represented the only instance of a 4‐way comparison. All other comparisons were between a single species at two different times. We conducted a Procrustes ANOVA among each genus to determine the effect of each variable, as well as interaction terms, on shape [e.g., (shape ~ centroid size * year)]. Both *R* ^2^ and *Z*‐scores were used to assess and compare variables and interaction terms. In all analyses, the null model consisted of shape and the effect of size alone (shape ~ centroid size), while the full model also included the date collected as the independent variable (shape ~ centroid size + year). In this way, we are able to evaluate the effects of year on shape while also accounting for any effects that may be associated with allometry. The only exception being in *Cichla,* where the full model used the interaction term species:year in place of year alone. All statistical tests, when appropriate, utilized a randomized residual permutation procedure (RRPP), ultimately subjected to 10,000 random permutations, and were done using GEOMORPH (Adams, Collyer, & Kaliontzopoulou, 2018; Adams, Collyer, & Sherratt, 2015) in R (R Core Team, 2018). RRPP is a method that randomizes the residual values from a null model to compare the statistics associated with a full model (Collyer & Adams, 2007; Collyer, Sekora, & Adams, 2015). We also performed tests of morphological disparity (i.e., variation in geometric shape) utilizing Procrustes variances (Collyer, Sekora, et al., 2015). For the purpose of significance testing, α = 0.05 was used for all analyses.

For visualization of our data, we performed a principal component analysis (PCA) on the allometry‐corrected Procrustes residuals. We generated convex hulls using the first three principal components to project the region of morphospace that each species:year group occupied. This not only allowed for us to visually assess the region of overlap between years within a species group, but also provided a visual representation of changes in shape that we observed in many of our groups.

## 3 RESULTS

### 3.1 *Cichla* species

For all species, we initially tested the effect of size, year, and the interaction of size with year, with the exception of *Cichla,* which contained a third variable distinguishing species. We then quantified both mean morphological shape and disparity within and between species collected before and after closure of the dam (Table 1, 2). An initial Procrustes ANOVA revealed that no interactions were meaningful and that the effect of year alone (*R* ^2^ = 0.069; *Z* = 4.25; *p* = <.0001) was comparable to or greater than the effect of size (*R* ^2^ = 0.088; *Z* = 4.20; *p* = <.0001) alone (Table S2). Predam species comparisons (predam *C. kelberi* vs. *C. pinima*) revealed no significant differences in shape (*p* = .3683) or morphological disparity (*p* = .9098). A postdam comparison showed a significant difference in the shape of *C. kelberi* and *C. pinima* (*p* = .0002), but not a difference in disparity (*p* = .5181). Across years and within species (*C. kelberi* predam vs. postdam; *C. pinima* predam vs. postdam), shape was significantly different for both species (*C. kelberi,* *p* = .0001; *C. pinima*, *p* = .0269). Further, the postdam collection showed significant increases in disparity for both species, *C. kelberi* (*p* = .0147, Procrustes Variance: predam = 0.00063, postdam = 0.00130) and *C. pinima* (*p* = .0593; Procrustes Variance: predam = 0.00066, postdam = 0.00116).

**Table 1:** Pairwise comparisons of shape between species:year groups

	*Cichla kelberi,* ≤1984	*Cichla kelberi,* 2018	*Cichla pinima,* 1987	*Cichla pinima,* 2018
*Cichla kelberi,* ≤1984	–	5.4862	0.2287	2.1289
*Cichla kelberi,* 2018	0.0001	–	6.1372	5.0857
*Cichla pinima,* 1987	0.3683	0.0001	–	2.2114
*Cichla pinima,* 2018	0.0307	0.0002	0.0269	–

Note

Effect sizes (*Z*‐scores) are above, *p*‐values below diagonal. (–) indicates diagonal.

**Table 2:** Pairwise comparisons of morphological disparity between species:year groups

	*Cichla kelberi,* ≤1984	*Cichla kelberi,* 2018	*Cichla pinima,* 1987	*Cichla pinima,* 2018
*Cichla kelberi,* ≤1984	–	0.00066	0.00004	0.00053
*Cichla kelberi,* 2018	0.0147	–	0.00064	0.00015
*Cichla pinima,* 1987	0.9098	0.0171	–	0.00049
*Cichla pinima,* 2018	0.0467	0.5181	0.0593	–
Absolute Variance	0.0006	0.0013	0.0007	0.0012

Note

Effect sizes (*Z*‐scores) are above, *p*‐values below diagonal. (–) indicates diagonal. Absolute variance for each group provided in the last row.

**FIGURE 3:**
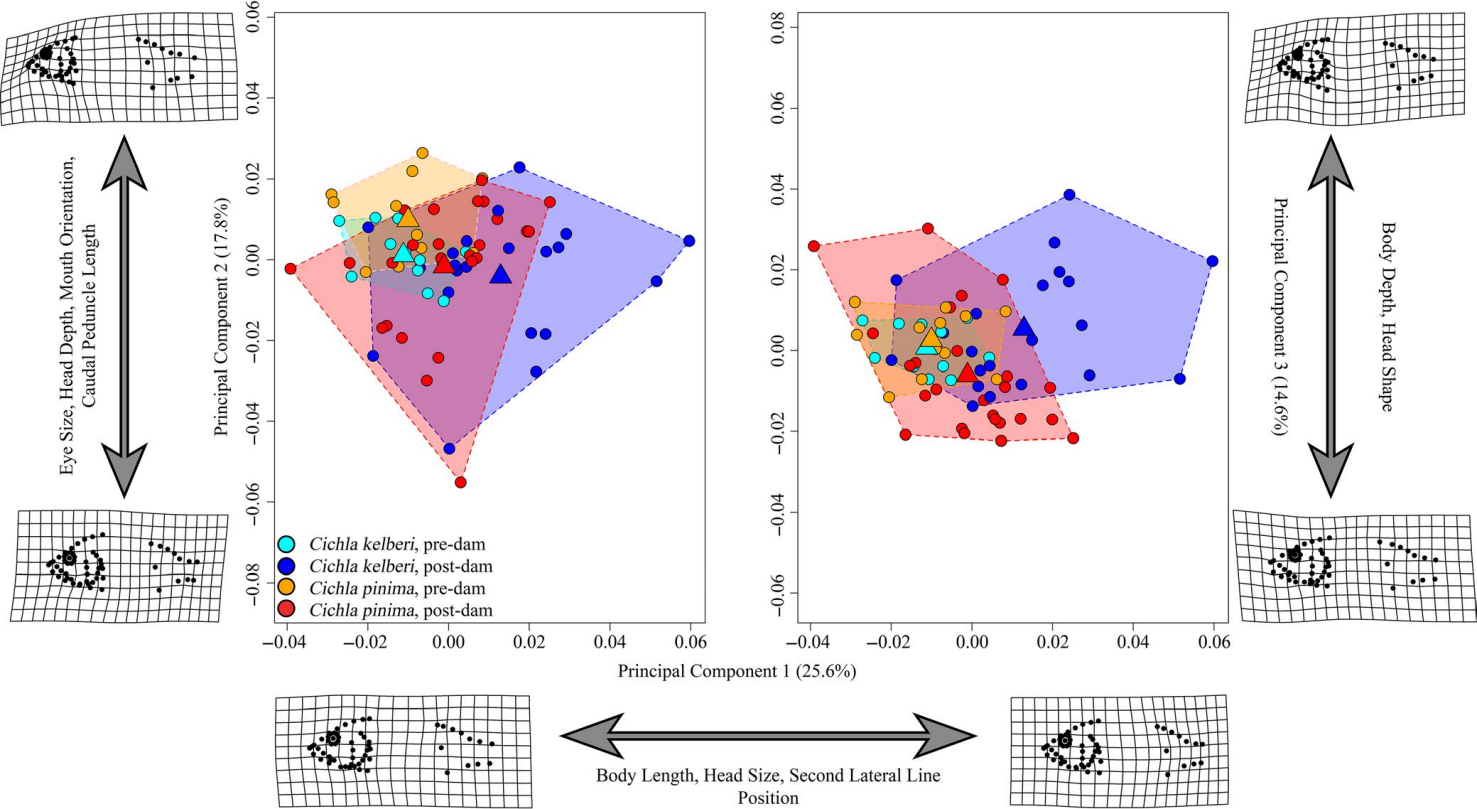
*Cichla* principal component plot (*X*‐axis = PC1 (25.6%), *Y*‐axis‐left = PC2 (17.8%), *Y*‐axis‐right = PC3 (14.6%)) indicating regions of morphospace occupied by each of the four species:year groups, as illustrated by colored convex hulls. Circles indicate individuals, and triangle represents the mean shape of each group within morphospace. Deformation grids are indicative of the minimum and maximum shape values for respective axes. Postdam collections showed significant increases in disparity for both species, *C. kelberi* (*p* = .0147, Procrustes Variance: predam = 0.00063, postdam = 0.00130) and *C. pinima* (*p* = .0593; Procrustes Variance: predam = 0.00066, postdam = 0.00116). Pre‐, postdam sample sizes: *Cichla kelberi*, *n* = 11, 21. *Cichla pinima*, *n* = 12, 25

Deformation grids of predam *Cichla* showed that both species were relatively long and possessed slender bodies. Predam specimens of *Cichla kelberi* possessed somewhat larger heads than their *C. pinima* counterparts, but differences were not significant. Deformation grids of postdam *Cichla* (Figure 3) illustrate morphological differences between the two species, with contemporary *C. kelberi* possessing deeper bodies and heads. This is in contrast to the elongated bodies and heads observed in postdam *C. pinima*.

The first three principal component axes (Figure 3) accounted for 58% of the total variance in shape. PC1 accounted for 25.6% of the variance. Negative scores along this axis were associated with elongated bodies, smaller heads, and a more anteriorly elongated second lateral line, while positive scores were associated with shorter bodies, relatively deeper heads, and a shorter second lateral line. Predam specimens exhibited more negative PC1 scores on average, whereas postdam specimens occupied the full range of this axis. PC2 accounted for 17.8% of the variance. Negative scores on this axis were associated with larger eyes, deep heads, superiorly oriented mouths, and short caudal peduncles. In contrast, positive scores were associated with comparatively narrow heads, terminally positioned mouths, and longer caudal peduncles. Similar to PC1, predam specimens were largely restricted to one end of this axis (i.e., positive PC2 scores), whereas postdam specimens spanned most of this axis of shape space. PC3 accounted for 14.6% of the variance and captured variation in body and head size. *Cichla* with negative PC3 scores had slender bodies and robust heads, while *Cichla* with positive scores can be characterized as having a deep body and narrow head. Mean shapes along this axis were largely similar between groups; however, postdam specimens occupied a greater amount of shape space. Contrary to our predictions, our results suggest that contemporary *C. kelberi* and *C. pinima* are not converging on a single “reservoir” ecomorph, but instead appear to be spreading into distinct regions of morphospace. We had predicted that both *Cichla* species would develop deeper bodies and larger mouths in response to the change to a lentic system. Our prediction was accurate only for *C. kelberi,* and opposite to what we observed in *C. pinima*, which appear to have developed more streamlined bodies over time. Further, predam specimens are highly constrained in morphospace on both PC1 axes and mean population shapes appear to be similar. However, postdam population means for both *C. kelberi* and *C. pinima* appear much how Kullander and Ferreira (2006) had described them with *C. kelberi* having a deeper body (~33% of its standard length) compared to *C. pinima* (~28% of its standard length) (Kullander & Ferreira, 2006). In more general terms, *Cichlia* species found in river systems tend to vary in head size and body depth, which may correspond to differences in microhabitat preference (e.g., fast flowing or more stagnant water).

Tests of allometry on geometric morphometric data failed to reject (*p* = .2412; Table S3) the null hypothesis that allometries were parallel, indicating that there has been no significant alteration of allometric trajectories over the past ~34 years since the Tucuruí dam was constructed. Across our four linear measurements, only two showed significant differences between species (Table S4). Both body depth and eye diameter differed between species, regardless of whether they were collected before or after the dam was closed. No significant differences were detected within species over time; however, trajectory analyses revealed significant differences in both jaw length and eye diameter. Slopes for *C. kelberi* (Figure S3) jaw length were significantly different (*p* = .0353), with predam specimens exhibiting accelerated jaw growth. Slopes also differed for eye diameter in both *C. kelberi* (*p* = .0013) and *C. pinima* (*p* = .0002), with accelerated eye size development in predam specimens. However, we note that size distributions for predam and postdam specimens were different, especially for *C. kelberi,* which suggests that differences in slopes should be interpreted with caution.

### 3.2 Geophagus neambi

An initial Procrustes ANOVA revealed that no interactions were meaningful and that the effect of year alone (*R* ^2^ = 0.114; *Z* = 3.87; <0.0001) was comparable to the effect of size (*R* ^2^ = 0.130; *Z* = 3.8781; *p* = <0.0001) alone (Table S2). *G. neambi* pre/postdam groups had significantly different shapes (*p* = .0003), but disparity was not different between the two groups (*p* = .1681; Procrustes Variance: predam = 0.0010, postdam = 0.0012). Deformation grids of mean shapes revealed that predam *G. neambi* have larger eyes and jaws, and more rounded preorbital region of the skull compared to postdam specimens. These data are consistent with our prediction that foraging generalists would be susceptible to anatomical change over time in this system.

**FIGURE 4:**
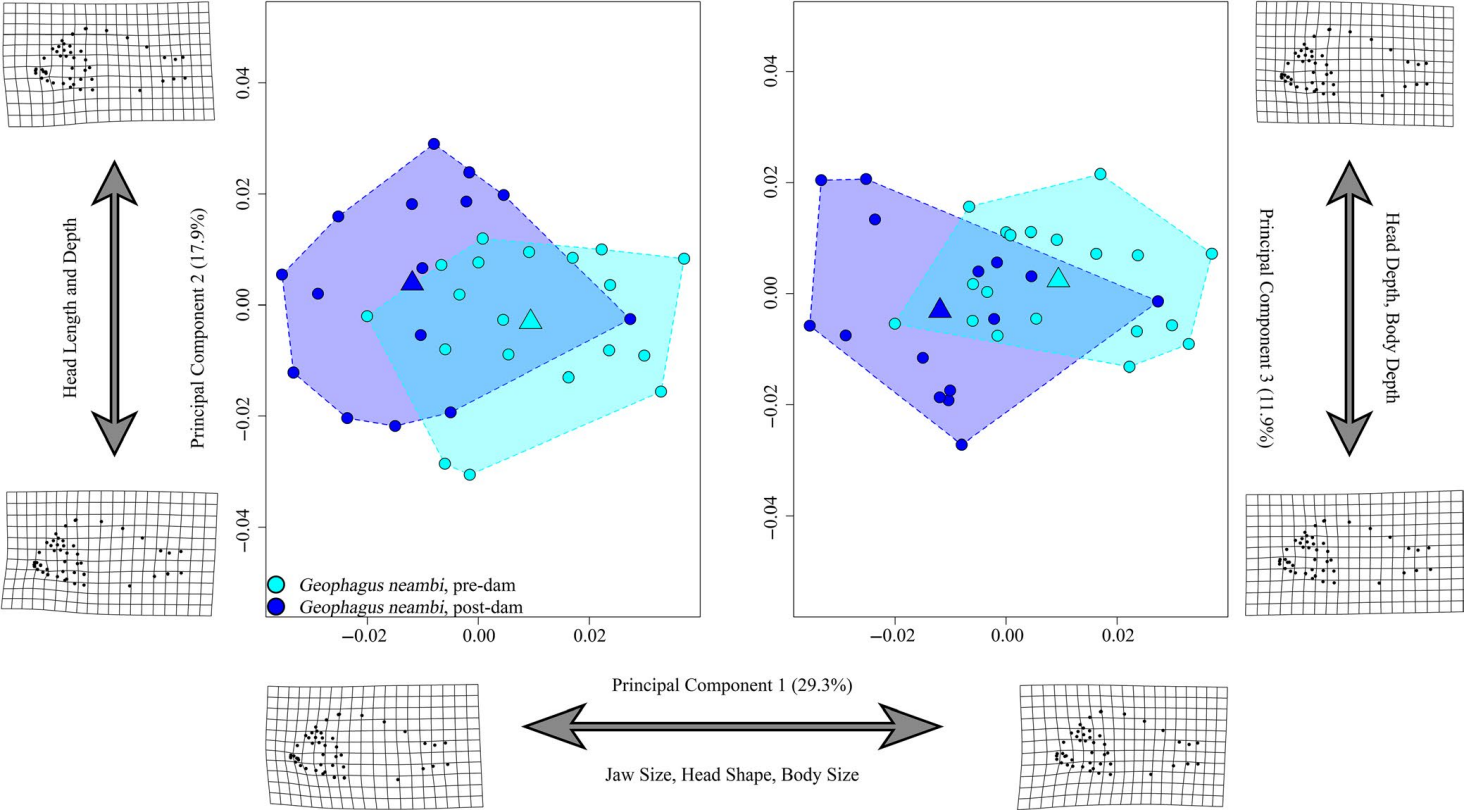
*Geophagus neambi* principal component plot (*X*‐axis = PC1 (29.3%), *Y*‐axis‐left = PC2 (17.9%), *Y*‐axis‐right = PC3 (11.9%)) indicating regions of morphospace occupied by each year group, as illustrated by colored convex hulls. Circles indicate individuals, and triangle represents the mean shape of each group within morphospace. Deformation grids are indicative of the minimum and maximum shape values for respective axes. Morphological disparity test reveals no significant (*p* = .1681) reduction in disparity between pre/postdam groups (Procrustes variance = 0.00096 and 0.00117, respectively). Pre‐, postdam sample sizes: *n* = 19, 15

The first three principal component axes (Figure 4) accounted for 59% of the total variance. PC1 explained 29.3% of the variance and was associated with relative head and body size and shapes. Negative PC1 scores represented relatively smaller jaws, acutely angled faces, and larger bodies. On average, postdam specimens exhibited negative PC1 scores compared to predam specimens. PC2 accounted for 17.9% of the variance and was primarily associated with variation in head size. *G. neambi* with negative PC2 scores possessed relatively short and deep heads. Pre/postdam specimens exhibited considerable overlap along this axis. PC3 explained 11.9% of the variance and captured variation in body depth and head profile, with negative scores associated with animals that possessed more shallow head profiles and less deep bodies. Similar to PC2, pre/postdam specimens exhibited overlapping distributions along this axis.

Tests of allometry on geometric morphometric data rejected (*p* = .0312; Table S3) the null hypothesis that allometries were parallel, suggesting that there has been an alteration of allometric trajectories over the ~34 years since the Tucuruí dam was constructed. Linear models on measurements revealed that body depth (*p* = .0120) and jaw length (*p* = .0284) were significantly different from predam *G. neambi* (Table S5). Predam *G. neambi* possessed both deeper bodies and longer jaws than the contemporary specimens. Neither caudal peduncle (*p* = .2983) nor eye diameter (*p* = .4967) was significantly different between the pre/postdam group. Slopes between pre/postdam (Figure S4) collections were significantly different for both jaw length (*p* = .0012) and body depth (*p* = .0049). In both cases, predam *G. neambi* experienced accelerated growth relative to their postdam counterparts. Similar to the *Cichla* data presented above, these data should also be interpreted with care. In this case, pre‐ and postdam specimens had similar size distributions, but size ranges were limited, meaning that slopes were highly dependent upon a handful of individuals at the periphery of the distributions. Given the current sampling scheme, interpretations of the size‐corrected linear measures should therefore be made with caution.

### 3.3 Satanoperca jurupari

Procrustes ANOVA revealed that no interactions were meaningful and that the effect of year alone (*R* ^2^ = 0.165; *Z* = 5.22; *p *= <.0001) was greater than the effect of size (*R* ^2^ = 0.088; *Z* = 3.70; <.0001) alone (Table S2). Pre/postdam groups had significantly different shapes (*p* = <.0001), but differences in disparity between the two groups were not significant (*p* = .1306; Procrustes Variance: predam = 0.0008, postdam = 0.0007). Deformation grids of mean shapes revealed conspicuous differences in head shape. In particular, predam *S. jurupari* possessed an elongated preorbital regions of the skull, upturned jaws, smaller eyes, and larger opercles, compared to postdam specimens. Differences were also apparent in the size and shape of the caudal peduncle. Similar to *G. neambi*, these data were consistent with our predictions with respect to trophic generalists.

**FIGURE 5:**
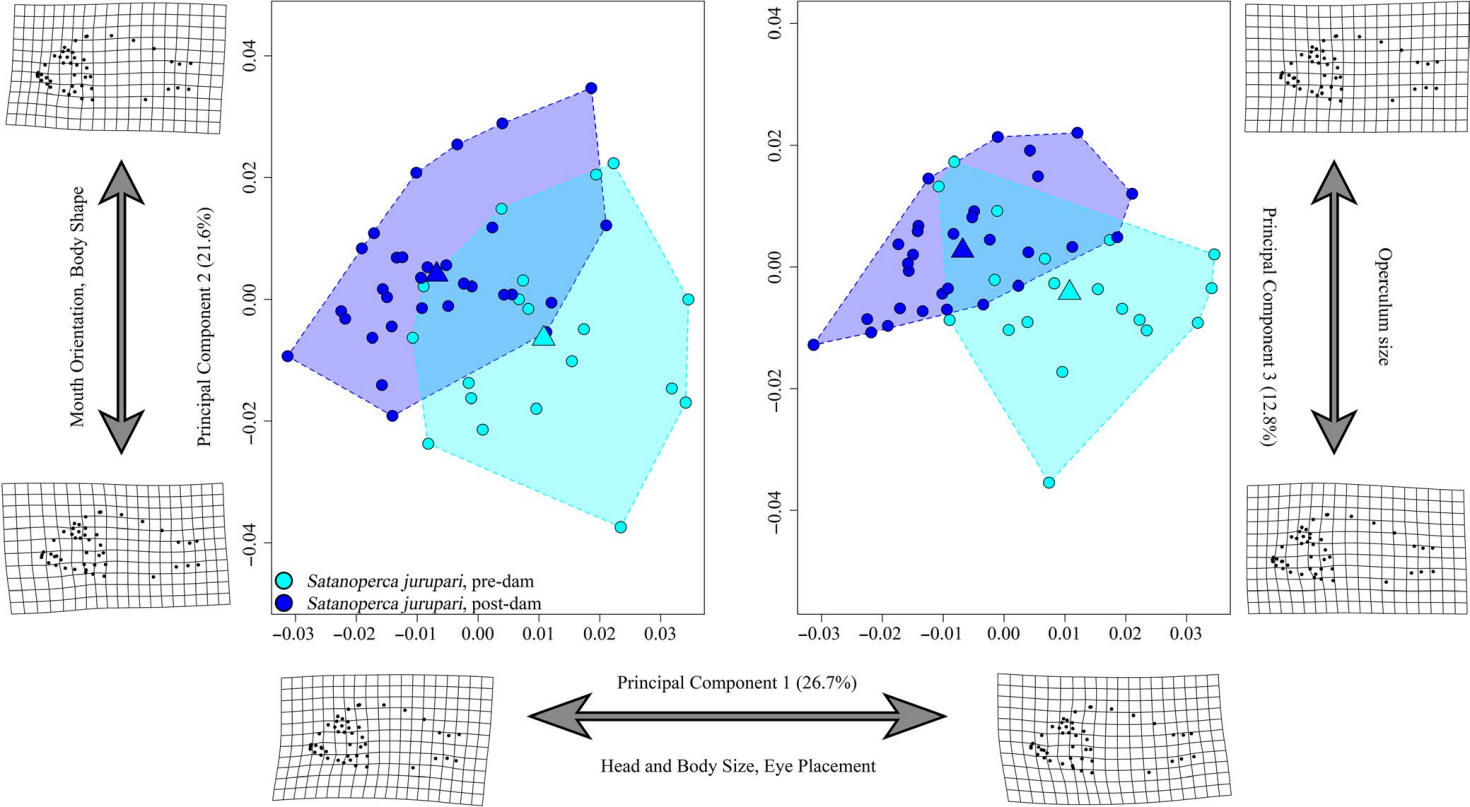
*Satanoperca jurupari* principal component plot (*X*‐axis = PC1 (26.7%), *Y*‐axis‐left = PC2 (21.6%), *Y*‐axis‐right = PC3 (12.8%)) indicating regions of morphospace occupied by each year group, as illustrated by colored convex hulls. Circles indicate individuals, and triangle represents the mean shape of each group within morphospace. Deformation grids are indicative of the minimum and maximum shape values for respective axes. Morphological disparity test reveals no significant (*p* = .1306) change in disparity between pre/postdam groups (Procrustes variance = 0.00084 and 0.00067, respectively). Pre‐, postdam sample sizes: *n* = 19, 30

The first three principal component axes (Figure 5) accounted for 61.1% of the total variance. PC1 accounted for 26.7% and described variation in head and body size, as well as eye placement. Negative scores were associated with short, rounded faces and more ventrally positioned eyes. Postdam specimens exhibited more negative PC1 scores on average. PC2 accounted for another 21.6% of the variance and described variation in mouth orientation and body shape. Negative scores were associated with a more terminally positioned mouth, and a slender body with a more pronounced posterior tapering toward the tail. In contrast, positive scores were associated with a more upturned mouth, and rounded body shape. Postdam specimens exhibited more positive PC2 scores. PC3 accounted for 12.8% of the variance and largely described variation in the opercular region of the skull. *S. jurupari* with negative scores tended to have larger opercles, whereas specimens with positive scores possessed smaller opercles. Postdam specimens exhibited slightly more positive PC3 scores on average.

Tests of allometry on geometric morphometric data failed to reject (*p* = .1026; Table S3) the null hypothesis that allometries were parallel, indicating that there has been no significant alteration of allometric trajectories over the past ~34 years since the Tucuruí dam was constructed. Across all four linear measurements, we observed no significant difference in absolute group means (Figure S4; Table S5). Additionally, we found no significant differences among slope trajectories in any comparison.

### 3.4 Caquetaia spectabilis

Procrustes ANOVA revealed that interaction between size and year was meaningful (*R* ^2^ = 0.056; *Z* = 2.71; *p* = .0051) and that, despite being significant, the effect of year alone (R^2^ = 0.040; *Z* = 1.84; *p* = .039) was less meaningful than the effect of size (*R* ^2^ = 0.167; *Z* = 4.49; <0.0001) alone (Table S2). Pre/postdam groups were significant at alpha = 90% in terms of both shape (*p* = .0935) and disparity (*p* = .0604; Procrustes Variance: predam = 0.0009, postdam = 0.0007). Deformation grids of mean shapes suggested that predam *C. spectabilis* possess slightly more upturned mouths, shallower bodies and anteriorly positioned eyes and pectoral fins compared to postdam specimens. Thus, while subtle differences were noted, predam and postdam *C. spectabilis* specimens were not as divergent as the two generalist species, which is largely consistent with our predictions.

**FIGURE 6:**
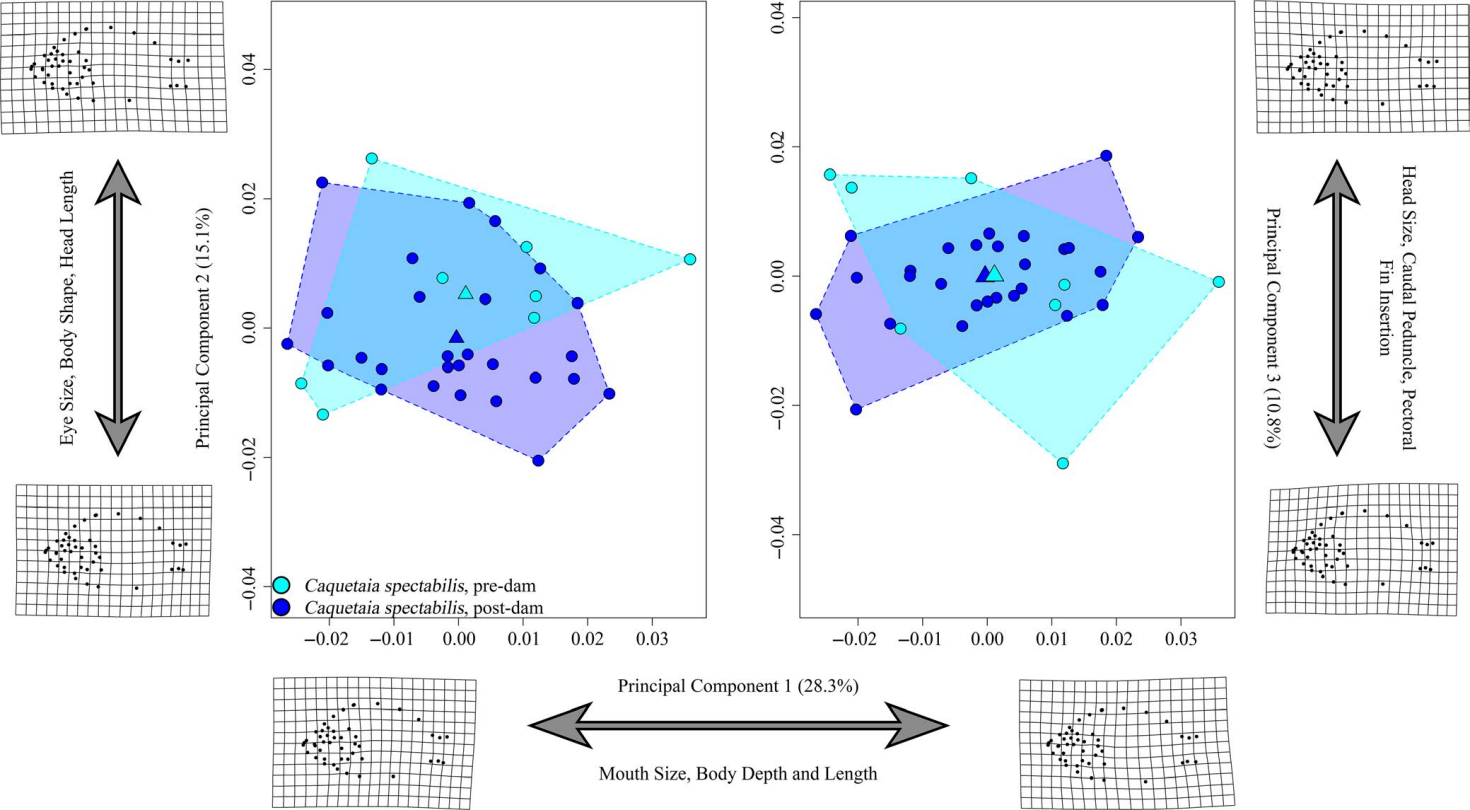
*Caquetaia spectabilis* principal component plot (*X*‐axis = PC1 (28.3%), *Y*‐axis‐left = PC2 (15.1%), *Y*‐axis‐right = PC3 (10.8%)) indicating regions of morphospace occupied by each year group, as illustrated by colored convex hulls. Circles indicate individuals, and triangle represents the mean shape of each group within morphospace. Deformation grids are indicative of the minimum and maximum shape values for respective axes. Morphological disparity test reveals a significant (*p* = .0604) reduction in disparity between pre/postdam groups (Procrustes variance = 0.00092 and 0.00067, respectively). Pre‐, postdam sample sizes: *n* = 8, 26

The first three PC axes (Figure 6) accounted for 54.3% of the total variance. PC1 accounted for 28.3% and was largely associated with differences in mouth orientation and body size. Negative PC1 scores were associated with a slightly shorter mouth and shorter, deeper bodies. The distribution of predam and postdam specimens overlapped along this axis. PC2 accounted for 15.1% of the variance and was predominantly associated with eye size and body shape and head length, with negative scores associated with relatively small eyes, short, rounded bodies, and shorter heads. Postdam specimens exhibited slightly more negative PC2 scores on average. PC3 accounted for 10.8% of the variance and was associated with variation in head and caudal peduncle size, as well as positioning of the pectoral fin base. Negative scores along this axis were associated with small heads and caudal peduncles, and a larger pectoral fin base. Predam and postdam specimens overlapped along this axis.

Tests of allometry on geometric morphometric data rejected (*p* = .0110; Table S3) the null hypothesis that allometries were parallel, indicating that there has been a significant alteration of allometric trajectories since the Tucuruí dam was constructed. No significant differences in group means were observed across all four linear measures (Figure S4; Table S5). Additionally, we detected no significant differences among slope trajectories in any comparison.

### 3.5 Heros efasciatus

Procrustes ANOVA revealed that interaction between size and year was not meaningful (*R* ^2^ = 0.027; *Z* = 2.88; *p* = .0045) and that, despite being significant, the effect of year alone (*R* ^2^ = 0.045; *Z* = 3.66; *p* = .0002) was less meaningful than the effect of size (*R* ^2^ = 0.439; Z = 5.72; *p *= <0.0001) alone (Table S2). Pre/postdam groups showed significant differences in both shape (*p* = .0103) and disparity (*p* = .0025; Procrustes Variance: predam = 0.00115, postdam = 0.00061). Notably, postdam specimens exhibited lower levels of morphological disparity than predam *H. efasciatus*. Deformation grids of mean group shapes showed that predam *H. efasciatus* have relatively larger eyes, smaller jaws, deeper heads and bodies, dorsal fin insertions behind operculum (postdam specimens appear to have dorsal fins that insert just above the operculum), and a shorter caudal fin base. These results were not consistent with our predictions regarding trophic specialists.

**FIGURE 7:**
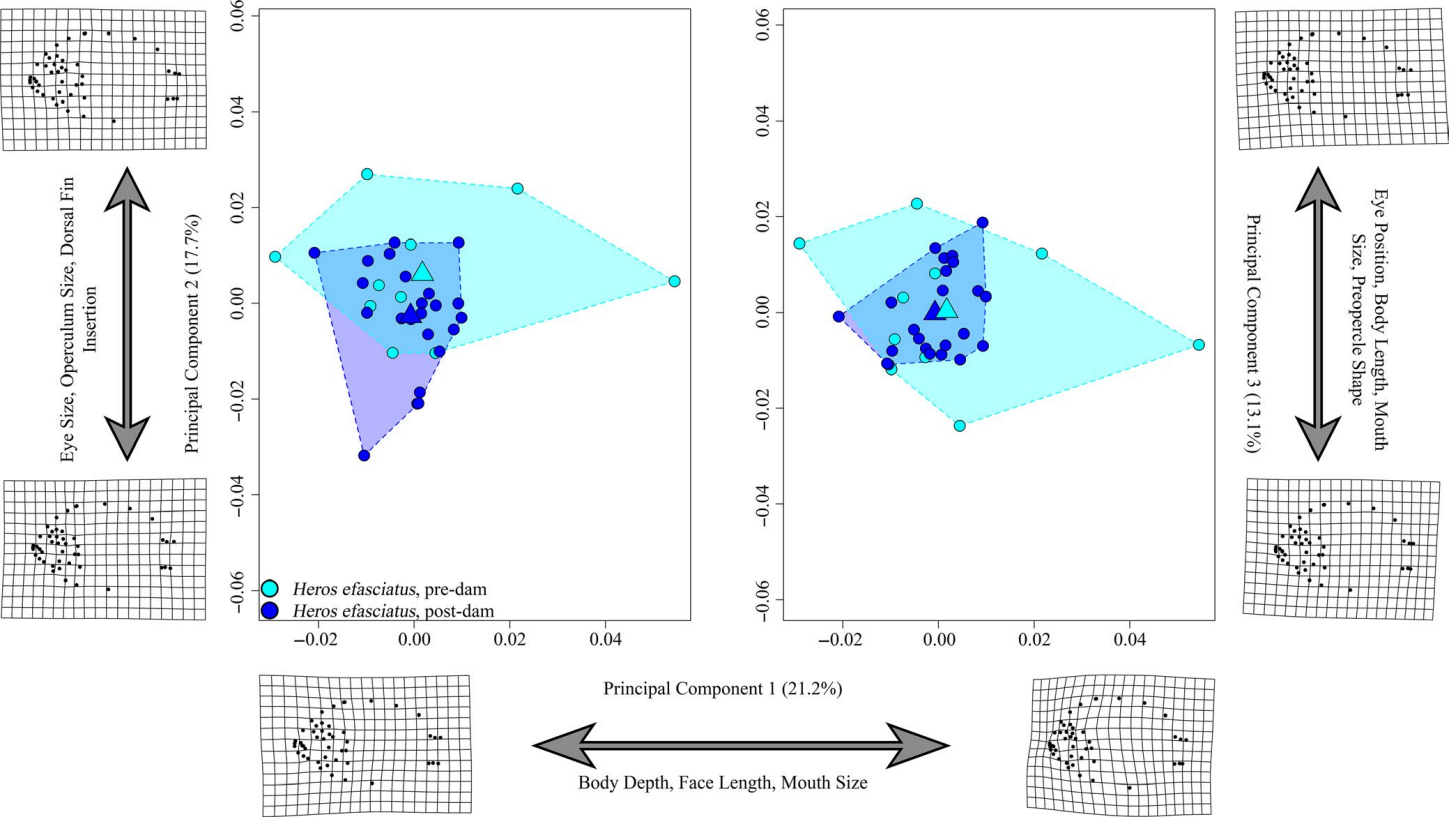
*Heros efasciatus* principal component plot (*X*‐axis = PC1 (21.5%), *Y*‐axis‐left = PC2 (17.9%), *Y*‐axis‐right = PC3 (14.5%)) indicating regions of morphospace occupied by each year group, as illustrated by colored convex hulls. Circles indicate individuals, and triangle represents the mean shape of each group within morphospace. Deformation grids are indicative of the minimum and maximum shape values for respective axes. Morphological disparity test reveals a significant (*p* = .0025) reduction in disparity between pre/postdam groups (Procrustes variance = 0.00115 and 0.00061, respectively). Pre‐, postdam sample sizes: *n* = 10, 23

The first three PC axes (Figure 7) accounted for 52.1% of the total variance. PC1 accounted for 21.2% and explained variation in head, mouth, and body size. Negative scores were associated with shallow bodies and longer faces. Postdam specimens were largely restricted to one end of this axis (i.e., negative scores on average), whereas predam specimens occupied the full range of PC1 scores. PC2 accounted for 17.7% of the variance and describes variation nape length, dorsal fin insertion, and eye size. Negative scores were associated with relatively smaller eyes and operculum, dorsal fin insertion just above the opercle (as opposed to positive scores that represent a more posterior dorsal fin insertion), and size of the pectoral fin base. Predam and postdam specimens overlapped along this axis but predam specimens trended toward more positive scores while postdam specimens trended toward more negative scores. PC3 accounted for 13.1% of the variance and was associated with eye position, body length, mouth size, and preopercle angle. Negative scores were associated with more ventrally positioned eyes, shorter bodies, and longer mouths, and a more rounded (as opposed to angular) preopercle. While both pre‐ and postdam specimens occupied a wide range of the observed PC3 scores, predam specimens had a somewhat larger occupancy, leaving postdam specimens to occupy a central position.

Tests of allometry on geometric morphometric data reject (*p* = .0045; Table S3) the null hypothesis that allometries were parallel, indicating that there has been a significant alteration of allometric trajectories. No significant differences in absolute group means were observed across all four linear measures (Figure S4, Table S5). Despite allometric differences in shape being detected, we found no significant difference among slope trajectories for our four linear measures.

## 4 DISCUSSION

The capacity of fishes to quickly respond to rapid environmental change, "natural" or anthropogenic, is well documented (Candolin, 2009; Franssen, 2011; Rüber & Adams, 2001). However, the present scenario offers a unique opportunity to explore how large‐scale environmental alterations can alter evolutionary trajectories and spur evolutionary divergence, especially in one of the most biologically diverse regions on the planet that is under increasing pressure from anthropogenic exploitation. The formation of the Tucuruí reservoir brought about dramatic changes that altered the local ecosystem from shallow, clearwater rapids to a deep, silty reservoir. While such operatic changes can be detrimental to fish populations (Agostinho et al., 2008; Alò & Turner, 2005; Fearnside, 2001), they can also spur instances of speciation (Dias et al., 2013). This study provides insight into not just how cichlid populations respond to large‐scale ecological changes, but also how different ecotypes respond to an altered landscape. We describe extensive morphological changes across the body for all cichlid species examined, regardless of ecotype, over the course of ~34 years following a major ecological change. While a subset of our data are limited to what is available from small historical collections, they ultimately suggest that cichlid species are experiencing rapid morphological change in response to large‐scale modifications of their environment. These findings are especially notable, as they stands in contrast to results recently presented by Geladi et al. (2019), which document little to no morphological change in characid species in response to damming. Thus, among two of the largest families of fishes worldwide, with especially high levels of biodiversity in the Neotropics, each appears to respond differently to major anthropogenic change, underscoring our limited ability to predict how different lineages will respond to similar ecological disturbances.

### 4.1 Anatomical changes are observed across cichlid ecomorphs

The peacock basses (e.g., *Cichla* species) are widespread throughout the Amazon basin, play important roles as large piscivorous predators (Fugi, Agostinho, & Hahn, 2001; Sharpe, De León, González, & Torchin, 2017; Williams et al., 1998), and are economically important for both food and recreation. Despite being native to the Amazon basin, these species have been widely introduced to numerous non‐native systems where they play prominent roles in the decline of native fishes (Kovalenko, Dibble, Agostinho, Cantanhêde, & Fugi, 2010; Sharpe et al., 2017).

The construction of the Tucuruí Dam, and subsequent formation of the Tocantins reservoir, has likely aided the establishment and success of local *Cichla* species, which have been reported to require stable water levels and adequate littoral zones for reproduction (Williams et al., 1998). *Cichla* are large bodied cichlids that are capable of traveling extremely long distances (Hoeinghaus, Layman, Arrington, & Winemiller, 2003), and have an evolutionary history largely shaped by the historical hydrology of South America (Willis, Nunes, Montaña, Farias, & Lovejoy, 2007). Our geometric morphometric data show that both *Cichla kelberi* and *C. pinima* appear morphologically similar in predam collections with semielongate bodies. They also exhibit indifferent disparities and are constrained to a relatively narrow region of morphospace (Figure 3). However, postdam specimens appear to have experienced changes in morphology that not only pushed the two species apart in morphospace and distinguished them from the historical collections, but also resulted in a near‐identical, twofold increase in disparity (Table 2). The divergence in shape that we observed in these two species may be due to the altered riverine state of the area and the introduction of a deep, lentic environment. It has been well documented that lentic environments promote the development of deep bodies, whereas lotic environments promote more streamlined body shape in fishes (Collin & Fumagalli, 2011; Gaston & Lauer, 2015; Geladi et al., 2019) that are more efficient in reducing drag when navigating such environments (Gosline, 1971). Mathematically, compressed, deep bodied objects (e.g., fish) should be less capable of navigating waters characterized by high flow, owing to increased drag (Batchelor, 1967; Lamb, 1975). This is conversely true for more streamlined shapes, which are predicted to generate less drag in fluidic space and are therefore more energetically efficient. This appears to be the case for one of our species, *Cichla kelberi*, where contemporary collections exhibit a relatively deep, robust overall body shape compared to predam specimens. In contrast, the local *C. pinima* population appears to have responded in the opposite way, exhibiting more streamlined bodies compared to historic collections. This apparent divergence in body shape between *Cichla* species is consistent with ecological character displacement, a hypothesis that may be tested in the future. Sympatric *Cichla* species have previously been shown to consume different ichthyofauna. In one study, *C. temensis* were found to consume primarily characid fishes, while sympatric *C. orinocensis* largely consumed other cichlids (Williams et al., 1998). Thus, the different reaction of these two species, *C. kelberi* and *C. pinima*, could indicate that niche space across the reservoir is being partitioned to reduce the effects of competition between two large, predatory fishes. In addition, differences in the growth trajectories of specific functionally relevant traits, such as eye and jaw size, are consistent with niche shifts over life history within the reservoir that are distinct from those in ancestral riverine populations.

We had predicted that generalists and specialists would respond differently to large‐scale ecological changes. While cichlids in general are renowned for their flexibility in foraging behavior (Hulsey et al., 2005; Liem & Osse, 1975), they may be arrayed along axes of ecomorphology related to foraging (Cooper et al., 2010; Hahn & Cunha, 2005; Wainwright, Osenberg, & Mittelbach, 1991). Accordingly, the four smaller species included here were chosen based on their placement along a continuum between foraging generalist and specialist. Consistent with our prediction, both generalist species, *Geophagus neambi* and *Satanoperca jurupari*, experienced significant deviations in shape but not disparity, effectively shifting position, but not distribution, in morphospace (Figures 4, 5). Both species experienced changes across the body, but the most striking shifts were largely associated with head morphology, including changes in eye size and placement, jaw length, skull dimensions, and opercle sizes. These shifts are predicted to be associated with alterations in feeding behavior and kinematics and are consistent with the observation that both *G. neambi* and *S. jurupari* have atypical diets within the reservoir (de Mérona et al., 2001). Notably, the anatomical changes documented here effectively make the two species appear more similar than they were historically. *G. neambi* developed smaller eyes, smaller jaws, and less rounded jaws, while *S. jurupari* developed larger eyes, a shorter preorbital region of the head, and more rounded jaws. Thus, *G. neambi* and *S. jurupari* appear to be converging on a common head shape, which predicts that similar feeding strategies are being employed by these two closely related species within the reservoir.

In contrast to our prediction, both specialist species, *Caquetaia spectabilis* and *Heros efasciatus*, also exhibited changes in body and head shape morphology. In addition, both appear to have experienced a reduction in morphological disparity. This could suggest that selective pressures experienced in the altered environment are working to canalize phenotypic variation in these species, generally reducing observed phenotypic variance. The fact that we saw relatively low R^2^ values and Z‐scores associated with mean shapes, but more obvious differences in disparity, supports the hypothesis that these two species (*H. efasciatus* and *C. spectabilis*) are experiencing phenotypic canalization centered around the mean shape of their respective populations. A similar trend in a reduction of morphological variation has been reported for *Cyprinella lutrensis* (Cypriniformes:Cyprinidae) in reservoirs when compared to stream dwelling populations (Franssen, 2011), as well as for characid fishes (Geladi et al., 2019).

### 4.2 Possible mechanisms of morphological change

While it is impossible to explicitly connect changes in cichlid morphology detected here to the construction of the Tucuruí dam, there are many reasons to assume that it has played an important role. For instance, it is well established that dam construction will introduce dramatic changes to the hydrology of the immediate area (Dugan et al., 2010; Maingi & Marsh, 2002; de Mérona, Vigouroux, & Tejerina‐Garro, 2005; Miller, 1961), thereby altering the ecology of residing taxa, and possibly creating a foundation for ecological opportunity by introducing novel ecological niches to the system (Losos, 2010; Schluter, 2000). Furthermore, species diversity has been shown to be reduced in this area following construction of the Tucuruí dam (dos Santos et al., 2004), which has likely opened up additional resources to the remaining species, possibly leading to new selection regimes (Lahti et al., 2009; Roughgarden, 1972).

A likely mechanism contributing to some of the changes documented here is phenotypic plasticity, which refers to the capacity for a single genotype to express various morphological, physiological, and/or behavioral phenotypes under different ecological conditions. Plasticity can allow populations to quickly adapt and persist when faced with new ecological challenges (Ghalambor, McKay, Carroll, & Reznick, 2007), including the formation of a large reservoir. The present study does not present information directly regarding plasticity in cichlids, but plasticity has been well documented in fishes (Alexander & Adams, 2004; Garduño‐Paz, Couderc, & Adams, 2010; Lema & Nevitt, 2006; Lindsey, 1981; Robinson & Parsons, 2002), and is especially notable within the feeding apparatus (Alexander & Adams, 2004; Parsons et al., 2016; Wainwright et al., 1991). Furthermore, plasticity in body and head shape has been well documented in cichlids (Chapman, Galis, & Shinn, 2000; Muschick et al., 2011), including Geophagini cichlids (Wimberger, 1991, 1992). An important topic for future investigation will be to determine the extent to which phenotypic plasticity has contributed to shape differences documented here. Such insights will not only inform a better understanding of short‐term adaptation to novel environments, but they may also contribute to an understanding of longer‐term patterns of evolutionary divergence. This is because plasticity has the capacity to influence longer‐term patterns of evolution by biasing the phenotypic variation that is exposed to selection (West‐Eberhard, 1989, 2003). Thus, by recapitulating a river‐to‐lake transition that has occurred repeatedly across many fish lineages, including cichlids, the Tucuruí system may provide insights into how plasticity has contributed to the earliest stages of adaptive radiations, a topic that has largely been a matter of theory and deduction.

Genetic mechanisms can also underlie short‐term changes in phenotype, mainly via the sorting of ancestral alleles. For instance, selection may act against alleles that are maladapted to the new lacustrine system, resulting in a different distribution of phenotypes in the new environment (Yoder et al., 2010). Interspecific hybridization can further alter the genetic pool through the random sorting of alleles, leading to the development of novel phenotypes (Salzburger, Baric, & Sturmbauer, 2002; Smith, Konings, & Kornfield, 2003). Transgressive segregation is an especially potent mechanism, whereby novel combinations of alleles in a hybrid population can lead to the expression of extreme phenotypic variation within just a couple of generations (Bell & Travis, 2005). Therefore, hybridization, coupled with ecological change not unlike that observed in the Tucuruí region, has the potential to lead to conspicuous shifts in morphology in a brief period of time. Morphological trends in *Cichla* are consistent with these factors, including a divergence in shape over time, as well as a drastic increase in morphological disparity. Changes in ecologically relevant shape are consistent with a shift in niche space, while expanded disparity suggests that *Cichla* species within the Tucuruí reservoir may be hybridizing. Indeed, many of the diagnostic characteristics of *Cichla* species were unreliable for our contemporary collections, including traditional meristics, coloration, and lateral line patterning. Approximately 10% of *Cichla* specimens collected from the reservoir in 2018, exhibited a mosaic of features, and were excluded from our analyses due to the inability to properly key them to species. Contemporary *Cichla* collections overlap with historical collections in shape space; however, the expansion of variation that we observe in *C. kelberi* and *C. pinima* is consistent with transgressive segregation, and is similar to documented cases of this phenomenon in East African cichlids (Albertson & Kocher, 2005). By acting on standing genetic variation, selection has the capacity to quickly alter patterns of phenotypic variation (Collin & Fumagalli, 2011; Lande & Shannon, 1996; Smith et al., 2003) and is credited for contributing to ongoing species divergence in old world cichlid radiations (Burress, 2014; Kocher, 2004; Malinsky et al., 2018).

Finally, changes in growth trajectories can explain the emergence of new morphological forms, as well as increased morphological diversity (Collyer, Novak, & Stockwell, 2005; Mina, 2001; Réveillac et al., 2015; Simonsen et al., 2017). Since we did not set out to specifically address this question, our experimental design is limited with respect to these types of analyses (e.g., in general, our samples only included one life‐history stage—adult). In spite of this caveat, we observed significant differences in allometric trajectories, based on our geometric morphometric data, for three of our five genera (*Caquetaia spectabilis, Heros efasciatus,* and *Geophagus neambi*). Furthermore, for at least one linear measure, slopes were significantly different between pre‐ and postdam *Cichla* and *Geophagus*. For both geometric and linear traits, when differences were noted, the trend was consistent, with accelerated growth noted for the predam population relative to its postdam counterpart. This is apart from the global shape of *Geophagus neambi,* where postdam slope was steeper than predam *G. neambi*. We found almost no significant difference across our linear measures despite observing significant differences in shape. Because geometric morphometric data are measuring global shape change, comprised of relative (and often subtle) changes across the entire body, they are more sensitive to detecting mean differences than any single linear measure. They also enable intuitive interpretations of the results via deformation grids (Thompson, 1917). From these interpolations, we may identify which regions of the body are varying more than other regions. While it can be more difficult to ascribe specific functional relevance to these types of changes, as compared to the length of a lever arm, for example, they are biologically relevant and represent pertinent organismal change (Adams et al., 2004; Rohlf & Marcus, 1993; Zelditch et al., 2012). Taken together, the allometric deviations in postdam collections that we document here could be due to a number of ecological changes that reservoir formation has brought to the region—including changes to turbidity, primary productivity, reproduction and feeding strategies, community composition, and predator–prey dynamics. This will be an interesting line of future investigation.

As with most experiments, there are limitations to the conclusions that may be considered. Here, we relied heavily on historical collections, which were limited in terms of quality (inadequately preserved for geometric morphometric analyses), quantity, and size distributions. We acknowledge these shortcomings (e.g., in detecting differences in allometric trajectories of linear measures), but feel confident nevertheless in our overall interpretations, and our conclusion that cichlid species are undergoing rapid phenotypic change (in levels and patterns of variation) in response to the novel environment within the Tucuruí reservoir. How this has occurred remains an open question. As a first step, future research should aim to determine the extent to which the morphological changes documented here are genetically determined versus the product of phenotypic plasticity.

### 4.3 Predicting outcomes?

The construction of the Tucuruí dam has resulted in pronounced changes to the hydrology of the immediate area, establishing a foundation for ecological opportunity by introducing the local ichthyofauna to novel niches. Among those changes, the most obvious is the transition from a fast flowing riverine system, to a deep, stagnant reservoir, significantly altering the flow regime and hydrodynamics of the system. Morphological changes in fishes following such alterations in flow regimes have been recorded in other taxa following impoundment (Franssen, 2011; Franssen et al., 2013; Haas, Blum, & Heins, 2010; Kristjansson, Skulason, Noakes, & Kristjánsson, 2002; Perkin & Bonner, 2011), and these can mimic adaptive divergence from river to lake systems (Langerhans, Layman, Langerhans, & Dewitt, 2003). In particular, when examining adaptation from systems with high flow to a system with no/low flow, stereotypical morphological shifts are often cited (Webb, 1984), including changes in body depth (Brinsmead & Fox, 2002; Collyer, Hall, et al., 2015; Collyer et al., 2005; Cureton II & Broughton, 2014; Haas et al., 2010; Langerhans et al., 2003; Santos & Araújo, 2015), mouth orientation (Franssen et al., 2013; Langerhans et al., 2003), size and location of paired/medial fin attachment sites (Aguirre, Shervette, Navarrete, Calle, & Agorastos, 2013; Brinsmead & Fox, 2002; Cureton II & Broughton, 2014; Santos & Araújo, 2015), and eye size and placement (Aguirre et al., 2013). These shifts are almost certainly due to adaptations to novel flow regimes themselves, as well as to novel prey items that may be found within them. Notably, many of these changes are reported, in one or more species, here in our comparison between contemporary (reservoir) and historical (riverine) specimens from Tucuruí. Although the speed at which anthropogenic changes occur is much greater than what is typically witnessed in natural systems, it is possible that the two events can inform one another. For example, understanding the mechanisms that underlie adaptive radiations may allow investigators to predict the types of morphological changes that may arise in lineages subjected to impoundment, which in turn may be applied to develop a more holistic picture of the long‐term environmental consequences. In addition, knowledge gleaned from studying the immediate effects of damming may provide a glimpse into the early stages of adaptive radiations, a topic that is largely a matter of theory and speculation. Thus, it is important to note that several dams built in the Amazon Basin, like the Xingu and Madeira Rivers dams, should also adopt similar studies monitoring how the fishes will adapt to a new environment. The outcome could lead to a better understanding of how environmental changes acts through time and space over resilient species.

## 5 CONCLUSIONS

The significant changes to the Tocantins, a product of deforestation, large‐scale agriculture, and the construction of the Tucuruí Hydroelectric Dam, have altered the ecological dynamics of the systems culminating with the Tucuruí reservoir. The historic fast flowing, clearwater rapids have been replaced by an extensive, deep, lentic system with high levels of turbidity and sediment deposition. Using a combination of museum specimens prior to the construction of the dam and contemporary specimens collected 34 or more years after the reservoir had been established, this study shows that native cichlid populations have undergone dramatic morphological changes in a short period of time, likely as a result of the reservoir formation. The closure of the dam has already resulted in a dramatic reduction in ichthyofauna diversity (dos Santos et al., 1984, 2004), possibly reducing competition for remaining species that now have access to an expanded array of ecological niches. The construction of the Tucuruí Hydroelectric Plant has allowed for a unique opportunity to witness how fish populations respond to sudden, large‐scale alteration to an ecosystem. This study provides evidence that ecologically related traits are changing in response to hydrological alteration, changes that are not limited to generalists alone, but apparent to varying degrees in all taxa investigated. Given the high number of dams constructed in the Amazon, particularly on the Tocantins, this study adds to the growing literature of what we can expect to occur within resident fish communities, notably in one of the most diverse freshwater fish communities on Earth.

## AUTHOR CONTRIBUTIONS

CCF and RCA conceived the project. AA, CCF, and RCA collected, processed, and imaged specimens. MCG performed the morphological analyses and analyzed linear traits. MCG, CCF, and RCA interpreted the morphometric results. MCG and RCA interpreted results from linear measurements. MCG and RCA wrote the manuscript. All authors contributed to manuscript revisions.

## Supporting information

Appendix S1

Data S1

## Data Availability

All raw data (landmark and linear measures) are available as supplemental material.
