# Supplementary material for: Rapid morphological change in multiple cichlid ecotypes following the damming of a major clearwater river in Brazil
Source: Evol Appl. 2020 Sep 23;13(10):2754–71. doi: 10.1111/eva.13080 (PMC7691474; doi:10.1111/eva.13080)
Supplement: Supplementary file 1 — Appendix S1 [file EVA-13-2754-s001.pdf]

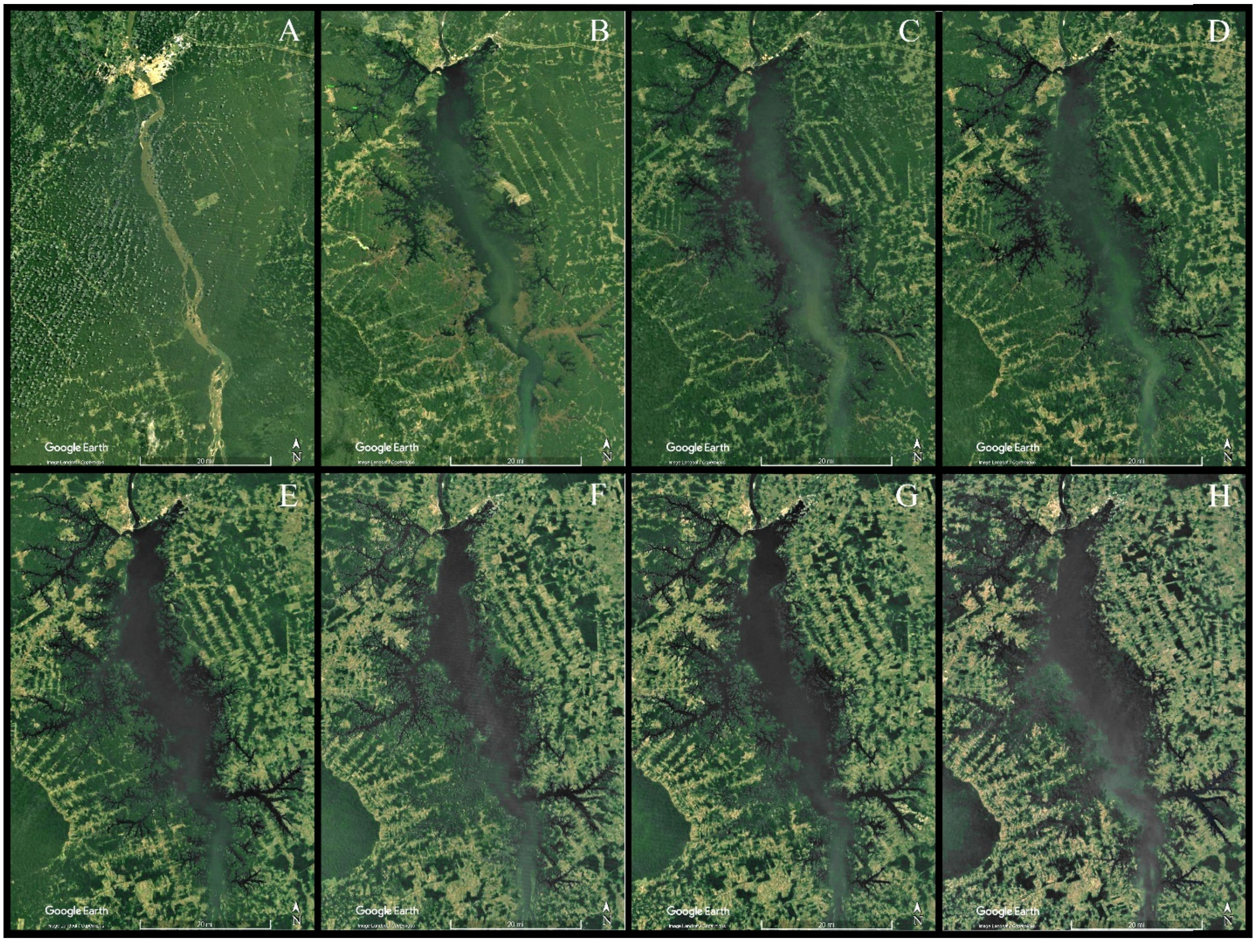

Supplemental Figure 1: Google Earth Landsat/Copernicus imagery of Tucuruí Hydroelectric dam vicinity over the past 32 years since closure: **A)** 1984, **B)** 1988, **C)** 1993, **D)** 1996, **E)** 2001, **F)** 2006, **G)** 2008, **H)** 2016. All images are taken to scale and scale bars represent ~32.2 kilometers (20 miles).

SUPPLEMENTAL TABLE 1. Museum accession numbers for all lots and specimens examined. Museum abbreviations are as follows, **MPEG**: Museu Paraense Emílio Goeldi; **MZUSP**: Museu de Zoologia da Universidade de São Paulo; **INPA**: Instituto Nacional de Pesquisas da Amazônia. **(n)** represents the number of specimens used against the number of specimens available in the lot. ∑ represents the combined size ranges from representative lots for pre- and post-dam specimens.

**Species**	**Lot #**	**Year**	**n**	**Size Range(cm)**
Cichla kelberi	MPEG 38519	2018	21/21	19.3 – 41.2
Cichla kelberi	INPA-ICT 24061	1980	2/2	
Cichla kelberi	MZUSP 46067	1970	1/1	
Cichla kelberi	MZUSP 50594	1970	1/1	
Cichla kelberi	MZUSP 50595	1970	2/2	
Cichla kelberi	MZUSP 50599	1970	3/6	
Cichla kelberi	MZUSP 63139	1970	2/2	∑ = 10.05 – 22.2
Cichla pinima	MZUSP 38409	1987	12/12	10.44 – 24.8
Cichla pinima	MPEG 38520	2018	25/25	16.39 – 33.49
Geophagus neambi	MZUSP 40927	1970	1/1	
Geophagus neambi	MZUSP 44835	1970	3/3	
Geophagus neambi	MZUSP 44954	1970	2/2	
Geophagus neambi	MZUSP 44956	1970	1/15	
Geophagus neambi	MZUSP 44958	1970	3/9	
Geophagus neambi	MZUSP 44959	1970	5/7	
Geophagus neambi	MZUSP 44965	1970	3/43	
Geophagus neambi	MZUSP 44966	1970	1/23	∑ = 8.37 – 13.65
Geophagus neambi	MPEG 36753	2018	8/15	
Geophagus neambi	MPEG 37099	2018	7/16	∑ = 8.63 – 14.02
Satanoperca jurupari	MZUSP 44194	1970	1/10	
Satanoperca jurupari	MZUSP 44217	1970	5/11	
Satanoperca jurupari	MZUSP 44233	1970	4/7	
Satanoperca jurupari	MZUSP 44234	1970	1/1	
Satanoperca jurupari	MZUSP 44235	1970	2/77	
Satanoperca jurupari	MZUSP 44236	1970	2/11	
Satanoperca jurupari	MZUSP 44237	1970	4/12	∑ = 9.46 – 15.52
Satanoperca jurupari	MPEG 38526	2018	30/30	11.25 – 17.92
Caquetaia spectabilis	MZUSP 45823	1970	1/5	
Caquetaia spectabilis	MZUSP 45824	1970	1/5	
Caquetaia spectabilis	MZUSP 45825	1970	2/8	
Caquetaia spectabilis	MZUSP 45827	1970	1/3	
Caquetaia spectabilis	MZUSP 45828	1970	1/2	
Caquetaia spectabilis	MZUSP 45830	1970	2/2	∑ = 8.68 – 15.07
Caquetaia spectabilis	MPEG 38517	2018	26/27	13.76 – 17.03
Heros efasciatus	INPA 9441	1980	9/9	

Heros efasciatus	INPA 9463	1980	1/1	∑ = 3.5 – 14.91
Heros efasciatus	MPEG 38525	2018	23/23	12.01 – 16.87

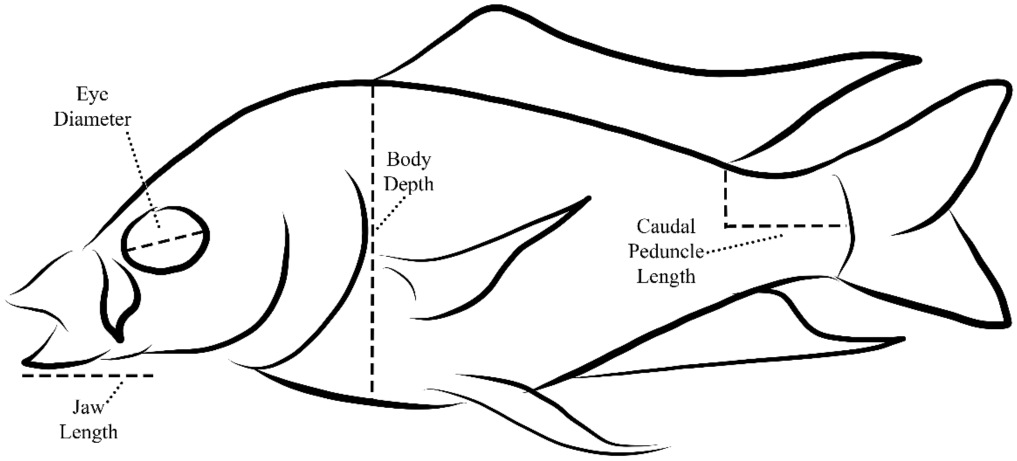

SUPPLEMENTAL FIGURE 2. Simple outline sketch of *Caquetaia spectabilis* detailing the four linear measurements taken across all cichlid specimens. Body depth was measured from the insertion of the spinous dorsal fin to the breast. Caudal Peduncle length was measured from the end of the soft dorsal fin to the end of the caudal peduncle proper. Jaw length was measured from the tip of dentary to the posterior margin of the articular. Eye diameter was measured as a straight line through the center of the orbit.

Supplemental Table 2. Results from ANOVA testing for effect strength on variables of interest and the interaction terms between them. For significance testing α = 0.05.

	R^2	F-Stat	Z-score	P
Cichla sp.				
Log(CS)	0.08813	7.2760	4.2025	<0.0001
Species	0.04312	3.5595	3.3165	0.0002
Year (Dam)	0.06908	5.0726	4.2535	<0.0001
Log(CS):Species	0.01805	1.4902	1.5486	0.0622
Log(CS):Year	0.00551	0.4551	-0.8932	0.8122
Species:Year	0.03000	2.4766	2.7724	0.0029
Log(CS):S:Y	0.00722	0.5931	-0.2583	0.5990
Geophagus neambi				
Log(CS)	0.13029	5.4467	3.8781	<0.0001
Year (Dam)	0.11415	4.7718	3.8720	<0.0001
Log(CS):Year	0.03794	1.5860	1.8546	0.0312
Satanoperca jurupari				
Log(CS)	0.08751	5.4065	3.6992	<0.0001
Year (Dam)	0.16451	10.1634	5.2245	<0.0001
Log(CS):Year	0.01960	1.2110	1.2853	0.1026
Caquetaia spectabilis				
Log(CS)	0.16748	7.0506	4.4890	<0.0001
Year (Dam)	0.04041	1.7014	1.8435	0.0385
Log(CS):Year	0.05576	2.3474	2.7149	0.0051
Heros efasciatus				
Log(CS)	0.43942	26.0597	5.7212	<0.0001
Year (Dam)	0.04470	2.6508	3.6592	0.0002
Log(CS):Year	0.02687	1.5938	2.8757	0.0045

SUPPLEMENTAL TABLE 3. Results from ANOVA, regressing centroid size against predicted shape (Shape ~ Centroid Size + Dam) across between pre/post dam genera. For significance testing, α = 0.05.

	R^2	P
Cichla	0.03317	0.2412
Caquetaia	0.0489	0.0110
Geophagus	0.0375	0.0312
Heros	0.02687	0.0045
Satanoperca	0.0196	0.1026

SUPPLEMENTAL TABLE 4. Results from Tukey Honest Significant Differences test on residuals of linear measurements (X ~ Standard Length) across four anatomically functional traits. Absolute differences in means above diagonal, p-values below. For significance testing, $\alpha = 0.05$.

	C. kelberi, pre	*C. kelberi*, post	*C. pinima*, pre	*C. pinima*, post
		Body Depth		
C. kelberi, pre	-	0.1408	0.6852	0.8418
C. kelberi, post	0.5942	-	0.5444	0.7010
C. pinima, pre	<0.0001	<0.0001	-	0.1566
C. pinima, post	<0.0001	<0.0001	0.4553	-
		Jaw Length		
C. kelberi, pre	-	0.0935	0.2618	0.0827
C. kelberi, post	0.8481	-	0.1683	0.0107
C. pinima, pre	0.1888	0.4412	-	0.1780
C. pinima, post	0.8804	0.9994	0.3595	-
		Caudal Peduncle Length		
C. kelberi, pre	-	0.0431	0.0665	0.1506
C. kelberi, post	0.9748	-	0.0234	0.1076
C. pinima, pre	0.9382	0.9954	-	0.0841
C. pinima, post	0.4371	0.5545	0.8205	-
		Eye Diameter		
C. kelberi, pre	-	0.0059	0.1906	0.1681
C. kelberi, post	0.9984	-	0.1846	0.1622
C. pinima, pre	<0.0001	<0.0001	-	0.0224
C. pinima, post	<0.0001	<0.0001	0.9140	-

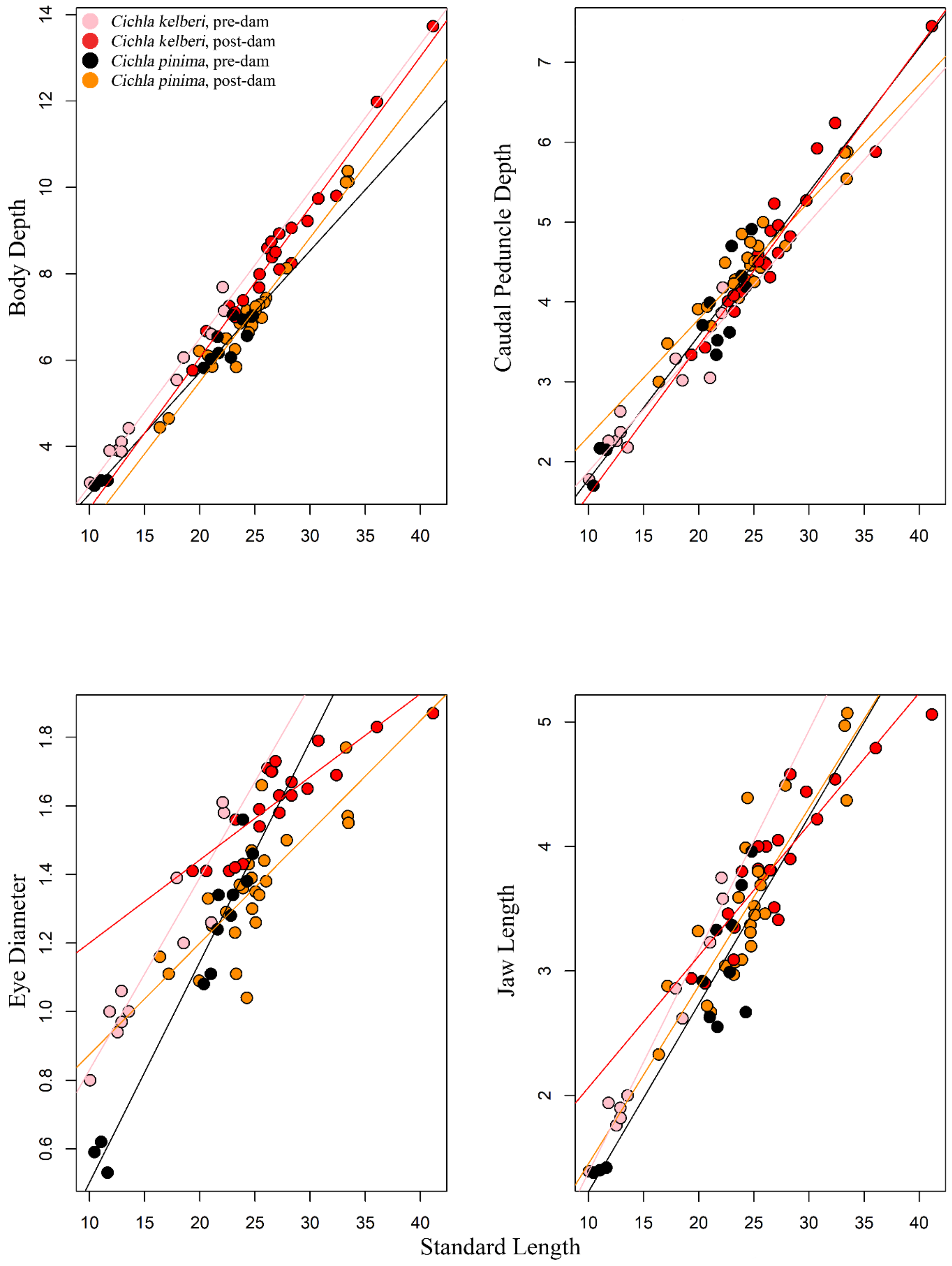

Supplemental Figure 3. Four traits of interest (body depth, caudal peduncle depth, eye diameter, and jaw length) regressed against standard length among the two *Cichla* species between the two year groups.

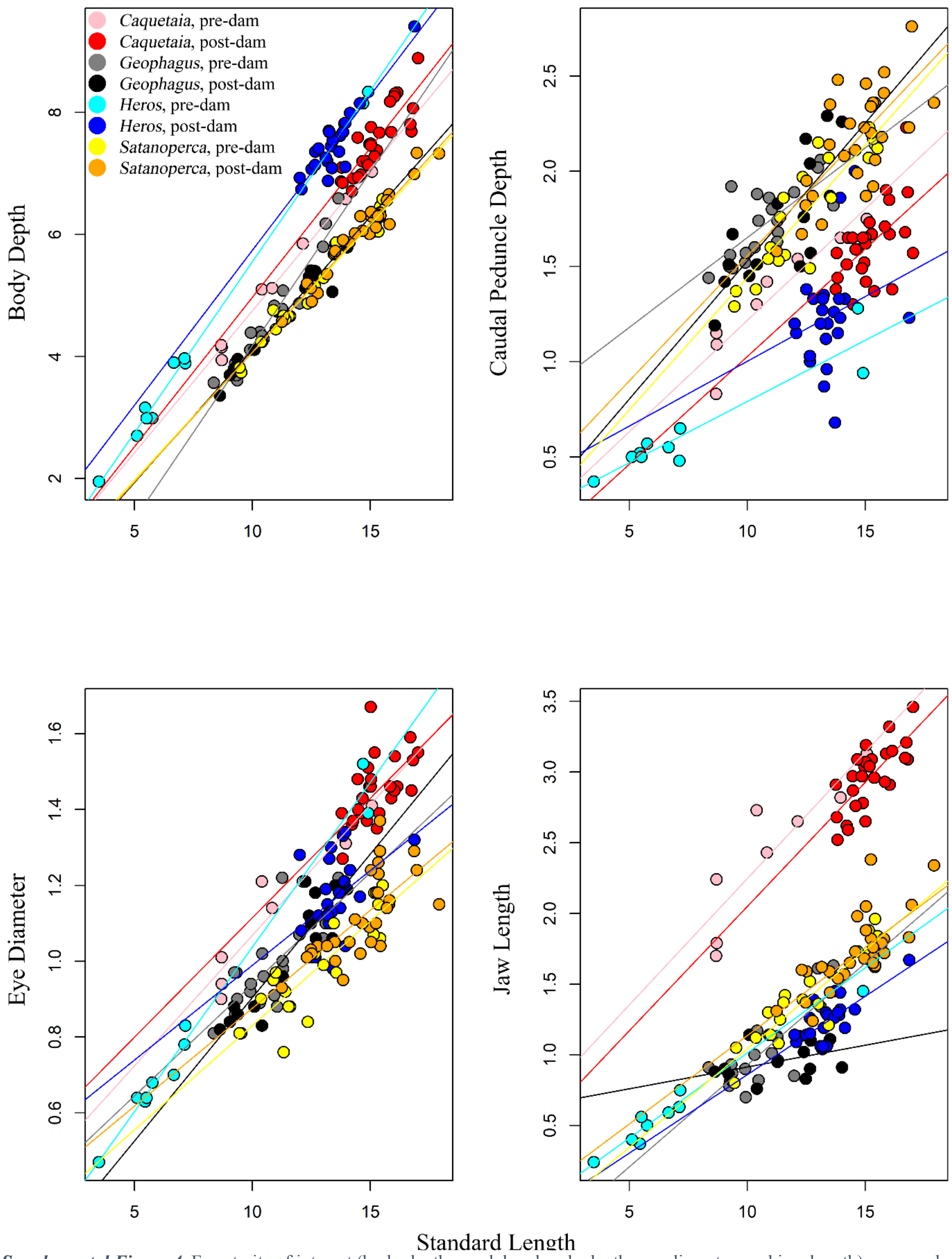

Supplemental Figure 4. Four traits of interest (body depth, caudal peduncle depth, eye diameter, and jaw length) regressed against standard length for the four remaining species between year groups..

SUPPLEMENTAL TABLE 5. Results from t- tests on residuals of linear measurements (X ~ Standard Length) across four anatomically functional traits. For significance testing, $\alpha = 0.05$.

	Absolute Difference in Means	P-Value
	Geophagus neambi	
Body Depth	0.2004	0.0120
Jaw Length	0.1274	0.0284
Caudal Peduncle Length	0.0665	0.2983
Eye Diameter	0.0162	0.4967
	Satanoperca jurupari	
Body Depth	0.0216	0.6838
Jaw Length	0.0287	0.5182
Caudal Peduncle Length	0.0618	0.2149
Eye Diameter	0.0243	0.2517
	Caquetaia spectabilis	
Body Depth	0.1195	0.1694
Jaw Length	0.0799	0.4129
Caudal Peduncle Length	0.0783	0.1938
Eye Diameter	0.0093	0.7161
	Heros efasciatus	
Body Depth	0.0173	0.7889
Jaw Length	0.0718	0.1185
Caudal Peduncle Length	0.0892	0.2141
Eye Diameter	0.0592	0.1084
